# Design, development and implementation of a digital burial record-keeping and management system in Bangladesh

**DOI:** 10.7189/jogh.15.04086

**Published:** 2025-02-28

**Authors:** Md Hafizur Rahman, AKM Tanvir Hossain, Uchchash Barua, Md Shahidul Islam, Ema Akter, Ridwana Maher Manna, Md Alamgir Hossain, Tasnu Ara, Nasimul Ghani Usmani, Pradip Chandra, Shafiqul Ameen, Sabrina Jabeen, Anisuddin Ahmed, Taufiq Zahidur Rahman, Mohammad Mamun-Ul-Hassan, Atiqul Islam, Beth Tippett Barr, Qazi Sadeq-ur Rahman, Shams El Arifeen, Aniqa Tasnim Hossain, Ahmed Ehsanur Rahman

**Affiliations:** 1 Maternal and Child Health Division, International Centre for Diarrhoeal Disease Research, Dhaka, Bangladesh; 2 Dhaka North City Corporation (DNCC), Kalachadpur, Gulshan, Dhaka, Bangladesh; 3 Nyanja Health Research Institute, Salima, Malawi

## Abstract

**Background:**

Digitalisation of death documentation in Bangladesh's graveyards is crucial for accurate mortality data and public health planning. Additionally, studying the usability, technology acceptance, and implementation aspects of the digital death record-keeping system, an innovative intervention that has not been previously explored, ensures the effectiveness, user adoption, and long-term sustainability. We designed, implemented, and evaluated a digital mortality surveillance system in graveyards in Dhaka city of Bangladesh.

**Methods:**

The study was conducted in six graveyards of the Dhaka North City Corporation (DNCC). First, we conducted formative research to understand the documentation process of the graveyard record-keeping and inform architecture for a digital record-keeping system. We then developed a digital record-keeping system for graveyard. A cross-sectional quantitative and qualitative study was conducted among web and app users, and graveyard managers and death records keepers.

**Results:**

A total of 200 app and web users and 14 record-keepers and managers participated. App and web users had high levels of system usability (88%) and found them easy to use (100%) and well-integrated (100%), with disagreement on complexity (100%), inconsistency (100%), and technical support needs (100%). These users had high technology acceptance and agreed the tools help accomplish tasks quickly (100%), improve performance (100%), and were easy to learn (100%). Digital death record-keepers reported using the system frequently (71%) but found it somewhat complex (14%) and not consistently easy to use (28%), with some reported that the systems were well integration (50%) and easy to learn (43%). While technology acceptance varied, death record keepers generally agreed that the system helps with quick task completion (50%) and performance improvement (57%), increase productivity (43%), enhance effectiveness (57%), and easy to use (50%). The digital death record-keeping system received mixed reactions, with younger, tech-savvy operators being optimistic, while older supervisors were sceptical and uncomfortable with technology. Additionally, the system was found to be feasible to an extent, particularly by data entry operators and graveyard managers.

**Conclusions:**

The digital death record-keeping system was generally well-received by app and web users for its usability and integration, though graveyard supervisor found it somewhat complex, with mixed reactions based on tech-savviness; overall, the system was deemed feasible and sustainable.

Births, deaths, marriages, divorce, adoption, and causes of death are the vital events which form the backbone of a nation's civil registration and vital statistics system. The meticulous recording of these events not only ensures the functioning of health care services but also fosters equity, empowerment, and heightened economic productivity within a society [1,2]. Among these events, the registration of deaths holds a paramount significance, aiding in estimating disease impacts, understanding causes of death, and thereby prioritising health strategies tailored to specific requirements [3].

In the context of Bangladesh, the necessity for accurate and current data on death rates has been acknowledged by the government. To meet this requirement, a comprehensive national civil registration system was initiated in 2004, further bolstered by the implementation of an online system in 2010 [4]. However, despite these efforts, challenges persisted. An evaluation in 2013 unearthed factors inhibiting the registration process, including insufficient awareness among health care staff regarding the importance of reporting deaths, limited public understanding of the significance of registration, complex registration processes, and inconsistencies between paper-based and electronic registration systems [5]. Particularly alarming was the revelation that 85% of deaths occurred at home without proper documentation [6], and those deaths occurring in private and public health care facilities were not consistently reported and monitored. Moreover, the causes of most of these deaths are not determined according to international standards as defined by the World Health Organization (WHO) [1]. This underscored the urgent need for improved tracking and notification systems to gain a more accurate understanding of the nation's mortalities.

The utilisation of data sourced from graveyards, cemeteries, and crematoriums is an innovative method for estimating population mortality rates in various countries, including Portugal [7], Indonesia [8], Iran [9], and Turkey [10]. These analyses involve studying patterns and spikes in burials to estimate mortality rates, particularly when official documentation is incomplete or unreliable. These studies confirmed that readily available digital death records have significantly contributed to understanding mortality rate dynamics compared to the cumbersome and time-consuming paper-based systems. Digitalisation facilitates the efficient extraction and analysis of information and increases accuracy in determining mortality rates, an essential component for public health planning and policy formulation.

In Bangladesh, where a significant number of deaths lack official documentation, graveyards, cemeteries, and crematoriums house valuable and unutilised data. This information is currently stored in comprehensive paper-based death records. By harnessing the potential of digitalisation, these resources could offer a more precise understanding of the nation's health. To address this, Dhaka North City Corporation (DNCC) and the International Centre for Diarrhoeal Disease Research, Bangladesh (icddr,b) have initiated a project to digitise death documentation in graveyards, improving the ability to track mortality, especially during the COVID-19 pandemic. The project aimed not only to estimate excess mortality due to COVID-19 but also to analyse disease patterns, identify emerging health issues, assess health care interventions, inform policy decisions, and allocate resources more effectively.

Previous digital graveyard systems have primarily concentrated on memorialisation, cultural heritage, public engagement with graves, and cemetery navigation [11–14]. However, no research has specifically examined the digitalisation of death records from graveyards, cemeteries, and crematoriums for real-time mortality tracking, early warning system, and public health purposes. The novelty of this study lies in its application of digital graveyard data for health surveillance in a context where traditional death reporting systems are incomplete. This method offers a pioneering approach to enhancing Bangladesh's mortality data infrastructure, with the potential to greatly improve public health outcomes through more timely and accurate death reporting. Here we share our experience in the design, implementation and evaluation of a digital death record-keeping and management system in graveyards in Dhaka city of Bangladesh.

## METHODS

### Study setting

We implemented the study in Dhaka North City Corporation (DNCC), which accommodates six million residents [15,16]. Under the jurisdiction of the DNCC, six Muslim cemeteries are administered, Uttara-4, Uttara-12, Uttara-14, Banani, Mirpur Buddhijibi, and Rayerbazar. The size of the graveyards varies from 8037 to 55 249 m^2^ (Appendix 1 in the Online **Supplementary Document**) and hold between 1155 (Uttara-4) and 70 196 graves (Rayerbazar). Each cemetery has maintained paper-based death registers from its establishment (**Table 1**). We selected these sites because they are managed by a single administrative entity (DNCC), which allowed for a consistent examination of the death documentation process under similar regulations and management practices. Furthermore, these cemeteries serve a diverse and densely populated urban area, making them representative of the challenges associated with death documentation in a large city context.

**Table 1:** Description of graveyards in Dhaka North City Corporation, 2023

	Uttara-4	Uttara-12	Uttara-14	Banani	Mirpur	Rayerbazar
Year Established	1994	2012	2019	1974	1983	2016
Earliest records	11 / 1994	07 / 2012	02 / 2019	04 / 1974	07 / 1983	03 / 2016
Grave capacity	1155	2027	1170	10 160	25 264	70 196
Recorded deaths	4756	2307	323	23 308	61 938	15 880

### Understanding the traditional documentation process

We conducted formative research to understand the documentation process of the graveyards. The formative research included on-site observations of death documentation process, in-depth interviews (IDI), electronic recording of the paper-based historical death records, and stakeholder consultation.

#### On-site observation

We conducted 12 on-site observations in six graveyards to examine the traditional death documentation process, including tools, methods, workflows, and documentation flows using a structured tool and checklist (Appendix 2 in the **Online Supplementary Document**). The observations were performed by a team of four experts with diverse backgrounds, including public health, social research, and digital health systems. Each graveyard was assessed by at least two experts to ensure inter-observer reliability and comprehensive coverage of the documentation process. We took detailed notes, photos, and identified all graveyard’s documentation materials including register book, burial application form, and supporting documents required from the relatives of the deceased for burial (Appendix 3 in the **Online Supplementary Document**).

#### In-depth interview

We conducted eight IDIs to understand the traditional documentation and record-keeping system using an unstructured qualitative data collection tool (Appendix 4 in the **Online Supplementary Document**). The interviews were conducted by two trained qualitative researchers with expertise in public health and social research, both holding backgrounds in anthropology and social science. One supervisor from each graveyard and two graveyard managers from DNCC participated in the IDIs. Prior to each interview, each participant was informed about the purpose of the study, their voluntary role, and the confidentiality measures in place. Written informed consent was obtained from all participants, and they provided explicit permission for audio recording. Each interview lasted approximately 20–30 minutes and was audio-recorded with the consent of the participants. The interviews were transcribed verbatim to ensure accuracy in capturing the participants' responses.

**Figure 1:**
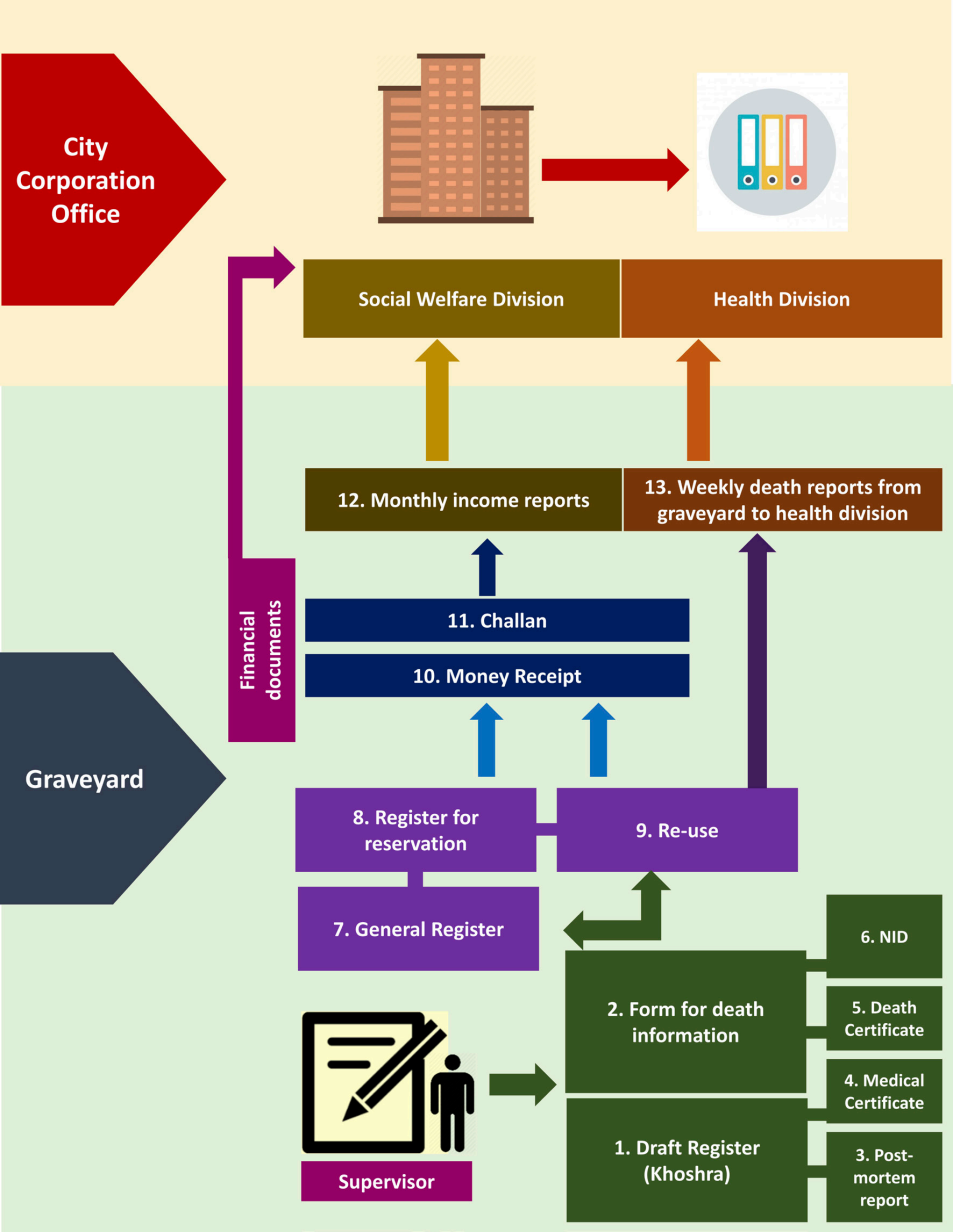
Paper-based data recording and reporting mechanism in the graveyards.

The data analysis was conducted using NVivo software [17], which facilitated the organisation and coding of qualitative data. The same two researchers who conducted the interviews also performed the analysis. The analysis involved a mix of inductive and deductive coding to ensure that both emerging themes and predetermined aspects of the documentation process were covered. We referred to the Consolidated criteria for Reporting Qualitative research (COREQ) guidelines to guide the reporting of our qualitative research processes [18]. Finally, we identified the data recording, documentation, and reporting mechanism in the graveyard by the on-site observation and IDIs (**Figure 1**).

#### Electronic recording of the paper-based historical death record

We took snapshots of all the death records in register books in six graveyards and electronically recorded the paper-based death records in an electronic record-keeping software developed by Java and .NET Core [19,20] (Appendix 5 in the **Online Supplementary Document**).

### Design of digital death record keeping system

We used participatory action research (PAR) to design graveyard’s digital death record keeping system architecture of death record-keeping system that met stakeholders' needs and addressed practical issues. The architecture of this system evolved through a dynamic, iterative process involving multiple stakeholders directly involved in the management and operation of graveyards.

#### Stakeholder identification and workshops

We initiated the design process by organising three stakeholder consultation workshops, bringing together six graveyard supervisors, four managers, and five IT specialists. These workshops were structured around a pre-defined agenda topic to guide the workshops and cover key aspects of the system design. The agenda topic guide included specific questions on operational workflows, reporting needs, integration with existing City Corporation data system, and technical feasibility. Graveyard supervisors provided operational insights. Managers offered a higher-level perspective, focusing on reporting needs, compliance with existing civil registration systems, and the broader public health goals that the system should support. IT specialists were integral in translating these requirements into technical specifications.

#### Mapping the requirements into system architecture

**Figure 2:**
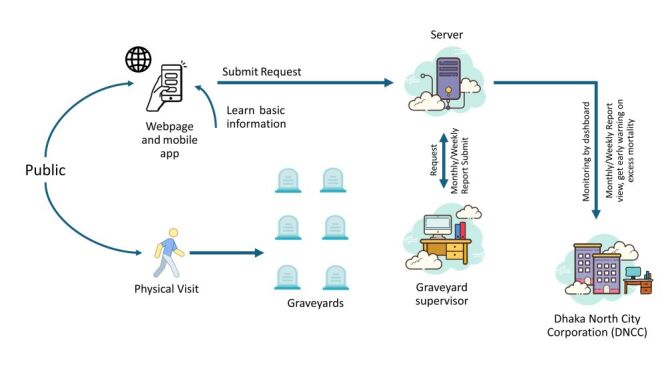
Architecture of the digital death record keeping and management system of the graveyards.

Based on the input from the workshops, we began to map out the system architecture. This process involved defining key components and their interactions, with the architecture following a modular design to ensure flexibility and adaptability (**Figure 2**).

#### Iterative prototyping and testing

Following the initial design, we developed a prototype and conducted testing sessions with stakeholders. This involved hands-on trials where graveyard supervisors and managers interacted with the system, providing feedback on usability, layout, and any technical challenges they encountered.

### Development

Following stakeholder consultations, architecture design, and prototype testing, we developed an integrated system for digital graveyard management (Appendix 6 in the **Online Supplementary Document**). This system serves multiple user groups:

1. General public: The mobile app (Appendix 7 in the **Online Supplementary Document**) and website (Appendix 7 in the **Online Supplementary Document**) were developed for the general public to facilitate grave reservations and provide information access.

2. Graveyard supervisors: the death record-keeping platform (Appendix 7 in the **Online Supplementary Document**) was developed for eight graveyard supervisors and four data entry operators to manage records related to burials and maintain up-to-date documentation.

3. DNCC managers: the dashboard, along with an early warning notification system (Appendix 7 in the **Online Supplementary Document**), was used by Dhaka North City Corporation (DNCC) managers to monitor graveyard operations and track excess mortality.

Additionally, we created digital maps of six graveyards using drone technology and developed a web application for kiosk systems at the graveyards (Appendix 7 in the **Online Supplementary Document**). The kiosk systems were intended for visitors to access grave information directly on-site.

### Implementation

We uploaded the mobile app to the Google Play Store and launched the website. From April to August 2022, we set up computer equipment in six graveyards (Appendix 6 in the **Online Supplementary Document**). Since the graveyard supervisors and managers lacked prior computer training, two developer team members invited all eight supervisors and two managers for training on basic computer skills and the digital record-keeping system, with sessions held twice a month for six months. Despite these efforts, three supervisors refused to participate in the training and adopt the system, Among the five supervisors received the training, one ultimately refused to adopt the system. Thus, four supervisors, two managers, and four computer operators adopted and used the system following the training.

### Study on system usability, adoption, and implementation experience

#### Study design

To understand the system usability, technology adoption, implementation experiences, we conducted both quantitative and qualitative studies. The primary research questions are outlined below in **Table 2**.

**Table 2:** Research questions of the study

Focus area	Research question	Indicators/themes	Outcome of interest
Usability	How usable and user-friendly is the system?	Use, complexity, ease of use, need of support, functions integrated, consistency, learnability, confidence in using.	System Usability Scale score, overall usability rating, system efficiency
Technology acceptance	How well is the system accepted by users?	Perceived usefulness, perceived ease of use, attitude toward using the system, behavioural intention to use, social influence, facilitating conditions, user experience	User acceptance, intention to adopt, perceived ease of use, perceived usefulness
Implementation experience	What are users' experiences with implementing the system?	Acceptability, adoption appropriateness, feasibility, barriers, sustainability	Acceptability, adoption rate, appropriateness of system, feasibility of integration, identification of barriers, sustainability

#### Quantitative assessment

A quantitative survey was conducted to assess usability and technology adoption among 200 users of mobile app and website, as well as among eight supervisors, four computer operators, and two managers involved in death record keeping and management systems. The questionnaire included sociodemographic variables like gender, age, years of education, marital status, household head status, number of children, smart phone use, operating system, frequency of use, house rental or ownership, and family death. Non-random convenience sampling was used to assess the System Usability Scale (SUS) and Technology Adoption Model (TAM). SUS and TAM details are presented in Appendix 8 in the **Online Supplementary Document**.

#### Qualitative assessment

Fourteen IDIs were conducted to understand acceptability, adoption, appropriateness, feasibility, barriers, and sustainability of graveyard digital death record-keeping system, each IDI among eight supervisors, four computer operators, and two managers. A semi-structured qualitative data collection tool was developed following the practical guideline of World Health Organization (WHO) on implementation research [21] (Appendix 9 in the **Online Supplementary Document**). Purposive sampling technique was followed to select respondents.

#### Analysis

Descriptive statistics including frequency and percentage was used to analyse the quantitative data. The interviews were transcribed verbatim to ensure accuracy in capturing the participants' responses. All Findings of the IDIs were analysed in NVivo to assess the documentation process [17].

## RESULTS

### Result from quantitative data

#### System usability among app and web users

**Figure 3:**
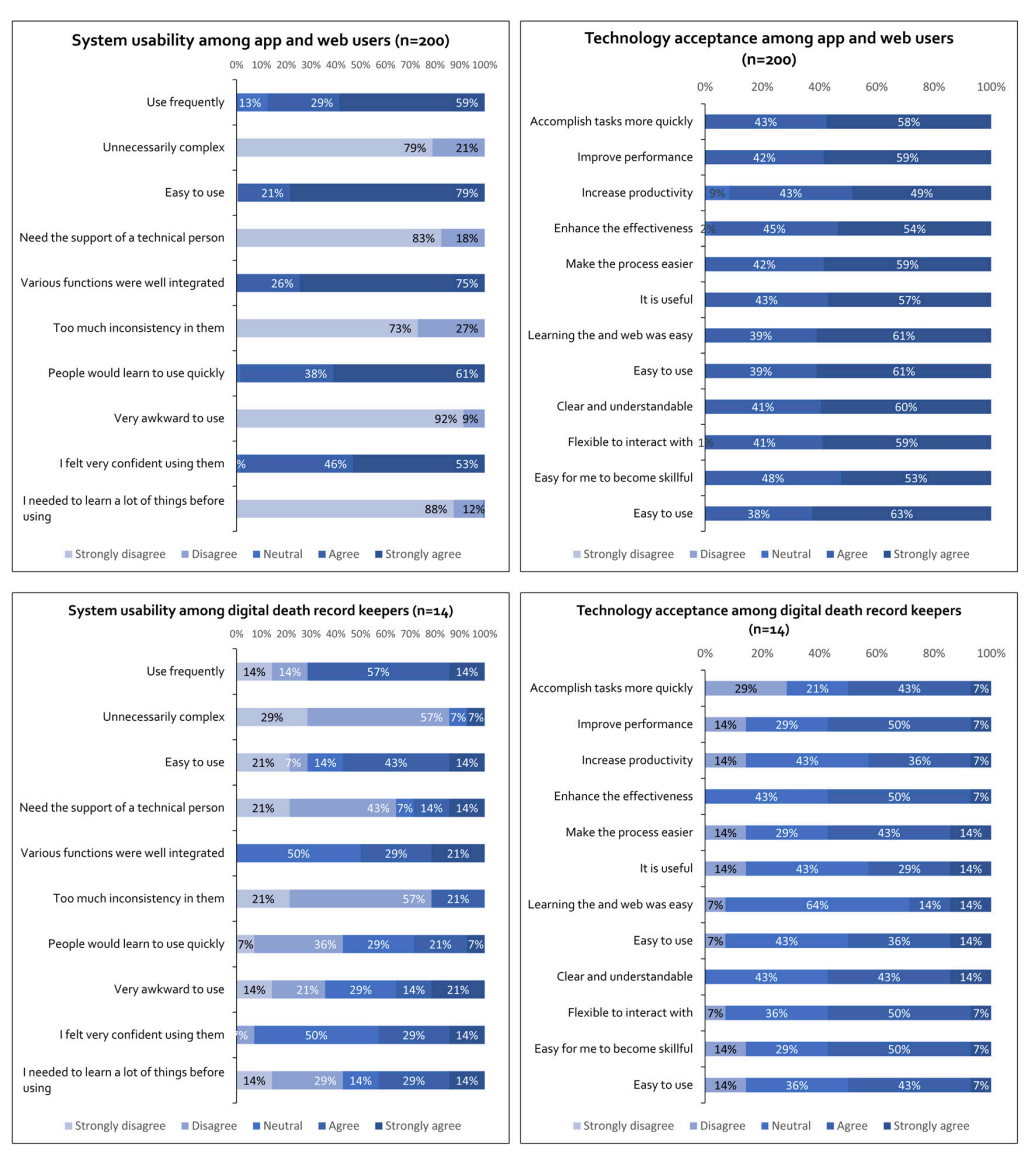
System usability and technology acceptance among the website, app users (n = 200), and digital death record keepers (n = 14).

A total of 200 app and web users took part in the system usability survey. Users majorly agree the system is easy to use (79%), well integrated (75%), and quickly learnable (61%). However, they majorly disagree with the system being unnecessarily complex (79%), needing technical support (83%), being inconsistent (73%), awkward to use (92%), and requiring much learning before use (88%). Users feel confident using the system (53%) and frequently use it (59%) (**Figure 3**).

#### System usability among app and web users

A total of 200 app and web users took part in the technology acceptance survey. Users majorly agree the system helps accomplish tasks more quickly (58%), improve performance (59%), increase productivity (49%), enhance effectiveness (54%), make processes easier (59%), is useful (57%), learning it was easy (61%), easy to use (63%), clear and understandable (60%), flexible to interact with (59%), and easy to become skilful (53%) (**Figure 3**).

#### System usability among graveyard record keepers

A total of 14 participants involved in graveyard record keeping took part in the system usability survey. Users majorly agree the system is used frequently (57%), not unnecessarily complex (57%), and does not need technical support (43%). They agree that various functions are well integrated (50%) and that there is too much inconsistency (57%). Users feel confident using the system (50%) and do not find it awkward to use (29%). They have mixed feelings about the system being easy to use, learning quickly, and needing to learn a lot before use (**Figure 3**).

#### System usability among graveyard record keepers

A total of 14 participants involved in graveyard record keeping took part in the technology acceptance survey. Users majorly agree the system enhances effectiveness (50%), makes processes easier (43%), is clear and understandable (43%), and flexible to interact with (50%). They also agree it improves performance (50%) and increases productivity (43%). However, users have mixed feelings about the system's ease of use, its usefulness, and becoming skilful quickly. Users somewhat disagree that the system accomplishes tasks more quickly (29%) (**Figure 3**).

### Implementation

The qualitative study explored acceptability, adoption, appropriateness, feasibility, barriers, and sustainability among eight supervisors, four computer operators, and two managers. **Table 3** provides the summary findings of the qualitative study.

**Table 3:** Key themes and summary of findings from qualitative interviews

Key themes	Summary findings
Acceptability	Younger, tech-savvy operators were optimistic.
	Older supervisors were sceptical and uncomfortable with technology.
Adoption	Four supervisors successfully adopted the system after training.
	Four supervisors resisted adoption, preferring paper records.
Appropriateness	Some found the system appropriate for improving accuracy and record management.
	Concerns over inadequate infrastructure and unsuitability for less tech-savvy users.
Feasibility	Data entry operators found the system feasible and timesaving.
	Supervisors struggled with technical issues and time required for data entry.
Barriers	Graveyard managers considered the system feasible, emphasizing its efficiency and practicality.
	Major barriers included lack of computer literacy and inadequate infrastructure.
	Cultural resistance to change and insufficient consultation with stakeholders.
Sustainability	Potential for sustainability with ongoing training and resources.
	Concerns about long-term support, high staff turnover, and continuous funding.
	Graveyard managers expressed optimism about the sustainability of the system, citing its value and their commitment to providing ongoing support.

#### Acceptability

Graveyard record keepers exhibited mixed feelings toward the digital system, primarily based on their age. The younger and more tech-savvy data entry operators found the digital system promising, while older supervisors were sceptical or uncomfortable with unfamiliar technology:

*Using a computer to record death information is a significant improvement. It reduces errors and makes data retrieval much easier* – Data entry operator 1, age 26

*This system has the potential to modernize our processes and make our work more efficient* – Data entry operator 2, age 32

*Paper-based records have worked for us for years. I don’t see why we need to change something that isn’t broken* – Graveyard supervisor 6, age 45

*I feel anxious using computers; it’s too complicated for me* – Graveyard supervisor 7, 57

#### Adoption

Four of the eight graveyard supervisors successfully adopted the system after receiving training. These individuals generally did not have prior exposure to technology; however, they were more open to learning new skills:

*The training was helpful. Although it still takes me a bit longer, I can now record information on the computer* – Graveyard supervisor 2, age 32

Despite training efforts, four supervisors resisted using the system. Resistance was often rooted in a lack of confidence and comfort with technology:

*I tried, but it’s just too complicated. I prefer the old way of doing things* – Graveyard supervisor 5, age 55

*I never grew up with computers, and it's hard to start now* – Graveyard supervisor 8, age 49

#### Appropriateness

Participants had divergent views on the appropriateness of the digital system given the existing infrastructure and operational context. Some participants found the system highly appropriate, especially in terms of improving accuracy, record management, and getting early warning notification on excess mortality.

*It’s more accurate and less prone to errors compared to our old paper records* – Data entry operator 3, age 48

*Having digital records helps us quickly find information when needed, and it became easier for us to check the weekly or monthly reports in few clicks and monitor the graveyards from office (DNCC). It is also made it easier for us to have early warning notification in our email when there are excess deaths in any graveyards…we can check and inform the health authority promptly* – Social welfare officer, age 40

However, others felt the system was not well-suited to their current environment. Some also felt that the system did not account for the realities of their work.

*My CPU (Central Processing System of computer) has not been working for a long time, I informed about it, but it took a long time to fix it. Meantime, I had to keep record in paper-based registers. Hence, it created a lot of back logs that I had to record in the digital system once the CPU was fixed* – Graveyard supervisor 2, age 32

#### Feasibility

Data entry operators and graveyard supervisors, who had better technical skills and interest, found the system feasible.

*Once you get the hang of it, it’s quite straightforward and actually saves time* – Data entry operator 1, age 26

*It streamlines our work, making it easier to manage large volumes of data* – Graveyard supervisor 4, age 36

Graveyard managers expressed confidence in the feasibility of the digital death record-keeping system. Additionally, they emphasised the importance of having the necessary infrastructure, such as computers and internet access, and existing resources such as office room and human resource to support the implementation of the digital system.

*The digital death record-keeping system has proven to be feasible for us. It streamlines our processes and allows for more efficient management of records… With the necessary infrastructure in place, such as computers and internet access, we find the system quite practical and easy to use* – Chief social welfare officer, age 50

However, for others, the system posed significant feasibility issues. The lack of consistent and reliable technical support was a major concern. Additionally, the time required to enter data was seen as a burden.

*Even after two years, it’s still hard for us. I think I take too much time in recording information of just one death. I too old for these technologies* – Graveyard supervisor 6, age 45

*We often face technical issues that we can’t solve on our own, and it disrupts our work* – Graveyard supervisor 2, age 32

*It takes much longer to enter data digitally than to write it down in our old logbooks* – Graveyard supervisor 3, age 40

#### Barriers

Several barriers impeded the effective adoption and use of the digital system. A significant barrier was the lack of computer literacy among many graveyard supervisors. The training provided was seen as insufficient by some.

*I never used a computer before this job, and it’s been very challenging* – Graveyard supervisor 7, age 57

*The training was too short, and we didn’t have enough hands-on practice* – Graveyard supervisor 5, age 55

Inadequate infrastructure was another major issue. Poor internet connectivity and security further complicated matters.

*Parts of the computers system such as the printer or the monitor sometimes get damaged, and it takes longer to fix it. This makes it difficult to maintain records efficiently* – Graveyard supervisor 6, age 45

*Sometimes, the server is sometimes down for days, making it impossible to update records in real-time. Besides, we the area is prone to thefts, and we feel insure with the computer system* – Graveyard supervisor 1, age 35

Cultural resistance to change also emerged as a barrier. Some participants felt that the digital system was imposed without sufficient consultation.

*We’ve been doing things the same way for decades. Changing to a new system is hard for everyone* – Graveyard supervisor 6, age 45

*We were not involved in the decision to switch to a digital system, and that has made it harder for us to accept it* – Graveyard supervisor 5, age 55

#### Sustainability

The sustainability of the digital system was a significant concern among stakeholders. Some participants saw potential for sustainability if certain conditions were met. Graveyard managers also expressed optimism regarding the sustainability of the digital death record-keeping system. They recognised the value of the system in improving the accuracy and accessibility of records, which contributed to their belief in its long-term sustainability. The data entry operators were optimistic.

*With regular training and supply of logistics, this system can be sustainable* – Social welfare officer, age 40

*As long as we have the necessary support and resources, I believe we can keep this system running smoothly* – Data entry operator 4, age 26

*We believe that with proper support and training, the digital system can be sustained in the long run. It has already shown its value in improving accuracy and accessibility of records. Our experience with the digital system has been positive, and we are committed to ensuring its sustainability by providing ongoing resources and support* – Chief social welfare officer, age 50

However, many participants were less optimistic about the long-term viability of the system. There was also scepticism about the commitment to ongoing funding and support.

*I do not feel motivated. I am not used to with computer. Without ongoing technical support and more resources, I don’t see how we can sustain this system* – Graveyard supervisor 7, age 57

*Initial enthusiasm is one thing, but will we have the same level of support and resources in five years?* – Graveyard supervisor 4, age 36

## DISCUSSION

The present study explores the implementation of Bangladesh’s first digital death record-keeping system, an innovative effort to address challenges in collecting accurate and timely mortality data. While Civil Registration and Vital Statistics (CRVS) systems are the most reliable sources for mortality data globally, the current CRVS system in Bangladesh remains incomplete and provides sub-optimal death registration coverage [1]. To compensate, the country has relied on alternative sources, such as surveillance systems and national surveys, which, despite their benefits, have notable limitations, including high costs, logistical challenges, and a lack of real-time data [22,23,24].

Given these limitations, there was a pressing need for an innovative and cost-effective alternative source of mortality data, especially during the COVID-19 pandemic, to understand effect of the pandemic on the overall mortality [25]. For example, similar to the present initiative, utilisation of graveyard or cemetery data to estimate mortality, especially during the COVID-19 pandemic has been evident in other countries like Portugal [7], Indonesia [8], Iran [9], and Turkey [10].

The study's results indicate differing perceptions of system usability and technology acceptance among web and app users compared to digital death record keepers. For web and app users, a strong agreement was observed regarding the frequency of system usage, ease of use, and integration of various functions. These findings suggest that the app and website effectively meet users’ needs and facilitates efficient information searching and retrieval processes. These results align with prior research emphasising the importance of user-friendly interfaces and seamless integration of functionalities in digital systems [26]. Moreover, the usability also may also be affected by user’s previous experience of using similar app or website. Existing studies has demonstrated that prolonged usage of a gadget by end-users leads to a perception of higher usability when they are already familiar with it [27,28]. Besides, device can play an important factor on usability. Utilising a single device for many purposes enables users to acquire expertise, hence enhancing their perceptions of usefulness [27,28].

The system usability and technology acceptance varied across different user groups. Web and app users found the system highly usable, frequently used it, and perceived it as easy to use with well-integrated functions. They did not find it complex or inconsistent and felt confident using it without needing extensive support. On the other hand, digital death record keepers had mixed feelings. While they agreed the system was frequently used and somewhat easy to use. However, the supervisors who refused training or did not adopt the digital system highlighted several barriers including lack of motivation, unfamiliarity with computers, limited involvement in decision-making, and insufficient training. Many found the transition from traditional methods too challenging without ongoing support, citing the complexity of the new system and the adequacy of paper-based records as reasons for their resistance. Future implementations should ensure that supervisors are actively involved in decision-making, receive thorough training, and have access to continuous support to ease the transition to digital systems.

In contrast, digital death record keepers expressed mixed views on system usability and technology acceptance. While some acknowledged the frequency of system usage, concerns were raised regarding the perceived complexity of the system and the time required to learn its functionalities. These challenges may stem from a lack of familiarity with technology and limited exposure to digital tools among certain user groups, as observed in our qualitative study. Among the 14 death record keepers, eight graveyard supervisors, did not have prior experience in using computers. Besides, three graveyard supervisors never used smartphone until the introduction of digital death record-keeping system. Average age of the graveyard supervisors was 42 years, and age of users may have a profound impact on system usability and technology acceptance [29,30]. Computer self-efficacy and anxiety can influence user’s acceptance of technology and perceived ease of use [31]. Previous studies reported that elder people has high fear of technology [32,33] and targeting underlying factors such as ensuing privacy, trust, and functionality may motivate to adopt technology and reduce technology related stigma and negative attitudes among older age people [33].

These findings underscore the importance of considering the age of the users and previous experiences of stakeholders in technology adoption processes [28,29]. The adoption of the digital system varied significantly across different roles and individuals, with successful adoption associated with adequate training and support. However, resistance to adoption was observed among some supervisors, highlighting the need for tailored training programmes and ongoing support mechanisms. Concerns were also raised regarding the appropriateness of the system given existing infrastructure limitations and the suitability of the system for less tech-savvy users. Additionally, in many LMICs, the cultural reality where seniority and rank are tied to older age means that younger juniors may not be able to question or disagree with their seniors [34]. This age-seniority dynamic can significantly impact the real-world feasibility of technology adoption, underscoring the necessity of aligning technological solutions with the specific needs, capabilities, and cultural contexts of end-users [35].

Graveyards, cemeteries, and crematoriums capture a significant proportion of urban deaths, yet their data remains largely untapped, locked away in non-standardised paper records. Previous digital graveyard systems have focused on memorialisation, cultural heritage, public interaction, and cemetery navigation [11–14]. However, no study has specifically explored the digitalisation of death records from these sites for real-time mortality tracking and public health purposes. This study is the first to digitalise graveyard death records in Bangladesh, enhancing mortality tracking through real-time, actionable data. The novelty lies in recognising these burial sites as valuable, often overlooked sources of public health data that can complement existing systems.

The innovation of this study is 2-fold. First, it seeks to review and evaluate the availability and usability of data from graveyards in Dhaka city. This is the first time a formal assessment has been conducted to determine whether such records can offer insights into mortality patterns and trends. Second, the study explores the feasibility of digitising the graveyard record-keeping system – a process that would not only improve the efficiency of operations at burial sites but also create a seamless flow of data into national health monitoring systems. This is a paradigm shift from traditional reliance on CRVS systems and surveys, offering a low-cost and sustainable solution to one of Bangladesh’s most pressing data challenges [30].

The study also takes a participatory approach by engaging key stakeholders, including graveyard supervisors, managers, and IT specialists, through a series of consultation workshops to design the architecture of the system. This ensures that the digital system design is user-cantered, responsive to real-world operational challenges, and adaptable to future needs. By integrating input from the people who manage and maintain these records, the system is tailored to meet both the operational needs of graveyard staff and the broader public health goals of mortality tracking. This co-creation process is another unique aspect of the study, helping to ensure long-term sustainability and adoption of the digital system [36].

The value of this study is its potential to transform a traditionally underutilised resource – graveyard records – into a vital tool for public health. The digitalisation of these records could have far-reaching implications for how mortality data are collected and used in Bangladesh. By providing real-time data on deaths, the system could offer immediate insights into mortality trends, allowing public health officials to respond swiftly to emerging health crises, allocate resources more efficiently, and tailor health interventions based on up-to-date information [37].

One of the most critical implications of this system is its capacity to serve as an early warning mechanism for excess deaths. In the event of a pandemic, epidemic or environmental disaster, the system can detect spikes in mortality far earlier than traditional methods, which rely on delayed or incomplete reporting. By monitoring sudden increases in the number of burials or cremations, public health authorities can be alerted to the possibility of an outbreak, environmental hazard, or other public health emergencies before they escalate [38]. This early detection capability is essential for reducing the impact of health crises by enabling swift government and health care responses, including targeted interventions and resource mobilisation [38].

For instance, during the COVID-19 pandemic, excess mortality – deaths above what would normally be expected – became a key indicator of the pandemic's true impact. However, reliance on national surveys or outdated records often meant these figures were only available months after the peak of the pandemic. A digital graveyard record-keeping system could help bridge this gap, providing real-time excess mortality data that would enable authorities to better understand the immediate effects of a crisis and plan accordingly. This can be critical for triggering emergency responses, such as increasing health care capacity, distributing medical supplies, or implementing public health measures, all of which hinge on timely and accurate data.

Moreover, the timeliness of this initiative cannot be overstated. The study was conceived and executed during the COVID-19 pandemic, a time when accurate mortality tracking became more critical than ever. The pandemic highlighted significant gaps in the reporting of deaths, particularly in urban areas, where overwhelmed health systems struggled to maintain accurate death registries [25,39]. This study responds directly to these gaps, offering a timely solution to improve mortality tracking not just during the pandemic but for future health crises as well.

In the long term, this project could also help Bangladesh estimate excess mortality, a key indicator of both the direct and indirect effects of health crises like COVID-19. By tracking patterns of death at the city level, it becomes possible to identify spikes in mortality that may indicate underlying health issues, environmental factors, or the spread of diseases. Furthermore, as a low-cost and scalable solution, this approach can be expanded beyond Dhaka to other cities and even rural areas, where similar systems could be deployed to provide comprehensive national mortality data. By documenting and sharing the design, implementation, and evaluation of the digital graveyard record-keeping system, this study serves as a blueprint for other countries facing similar challenges. It demonstrates how an often-overlooked source of data can be transformed into a powerful tool for understanding mortality trends, improving public health decision-making, and ultimately saving lives.

This feasibility study of a digital graveyard management system offers a novel approach to address challenges like the lack of accurate and accessible death records, crucial for public health monitoring and resource allocation. Digital systems can enhance data accuracy, reduce administrative burdens, and improve service delivery [40,41]. For instance, digital platforms facilitate real-time updates, advanced searches, and automated processes, ensuring record integrity. Integrating digital technologies provides insights into mortality trends, aids in early warning of excess deaths, and supports effective public health policies. This approach modernises graveyard management, aligns with global best practices, and contributes to digital transformation and smart city initiatives.

A project is under way in Bangladesh, in collaboration with the Dhaka North City Corporation (DNCC) and Local Government Engineering Division (LGED), to improve urban governance, develop GIS-based maps, and deliver smart services [42]. Our digitalisation of the graveyard death documentation process complements the ongoing Dhaka smart city initiative. However, feasibility challenges included technical skills, infrastructure support, and time constraints. Addressing these barriers required continuous training, logistical support, and infrastructure enhancements. These findings highlight the need for targeted training, infrastructure improvements, and stakeholder engagement [43]. Supervisors and managers indicated that the digital system would be sustainable due to long-term staff support, use of existing infrastructure, and alignment with the digital city initiative. To scale up the system nationwide, consideration must be given to the age and motivation of graveyard supervisors and death record keepers in adopting digital technology.

Our study had several strengths, including engaging stakeholders throughout the design and development of a digital death record-keeping system for graveyards in Dhaka, Bangladesh, and utilising various research methods, such as on-site observations, in-depth interviews, and stakeholder consultations. The study also integrated multiple platforms and applications to streamline graveyard management. However, there were some limitations. While we used the System Usability Scale (SUS) and Technology Acceptance Model (TAM) to evaluate usability and technology adoption, both tools have limitations. SUS offers a broad usability score but may lack the depth to capture specific user challenges, while TAM’s focus on perceived ease of use and usefulness might overlook other critical factors influencing technology acceptance in low-resource environments. Additionally, we lacked sufficient quantitative data to assess all implementation outcomes. As a result, we relied on qualitative research to evaluate aspects such as acceptability, adoption, and feasibility, which can introduce bias in interpreting results. This reliance on qualitative methods may limit the robustness of our findings, particularly regarding the generalisability of results to other settings like private graveyards in Bangladesh. Other challenges included sampling and selection bias, difficulties related to technology adoption in settings with diverse levels of computer literacy, and infrastructure and resource constraints, which could impact the long-term sustainability and effectiveness of the digital death record-keeping system.

## CONCLUSIONS

The digital death record-keeping system was found to be feasible to a certain extent. While it was appreciated for its potential to improve accuracy and efficiency, significant barriers in terms of adoption were identified. The key barriers included lack of computer literacy, inadequate logistic support, and the need for continuous technical support and training. To improve system usability and acceptance, especially among digital death record keepers, we recommend developing comprehensive training programmes, simplifying the user interface, and enhancing infrastructure to support effective use. Continuous technical support should be provided to address computer literacy challenges, and efforts should be made to integrate functions seamlessly and reduce inconsistencies. Regular performance monitoring will help identify areas for improvement, ensuring the system meets user needs effectively and provides comprehensive, timely data for public health action.

The global implications of this study are significant, especially for low- and middle-income countries (LMICs) facing challenges with incomplete and delayed mortality data due to underdeveloped civil registration systems. By demonstrating the feasibility of digitising graveyard records to capture real-time death data, this study provides a scalable, cost-effective model for mortality tracking that can be adapted to similar low-resource settings. By leveraging existing burial sites as data sources, LMICs can improve mortality surveillance, track and get early warning for excess mortality, inform public health strategies, and respond more effectively to emerging health crises, offering a replicable framework for strengthening health systems globally.

## Additional material


Online Supplementary Document
