# Supplementary material for: Design, development and implementation of a digital burial record-keeping and management system in Bangladesh
Source: J Glob Health. 2025 Feb 28;15:04086. doi: 10.7189/jogh.15.04086 (PMC11894443; doi:10.7189/jogh.15.04086)
Supplement: Online Supplementary Document [file jogh-15-04086-s001.pdf]

# APPENDIX1: SIX GRAVEYARDS UNDER THE ADMINISTRATION OF DHAKA NORTH CITY CORPORATION (DNCC)

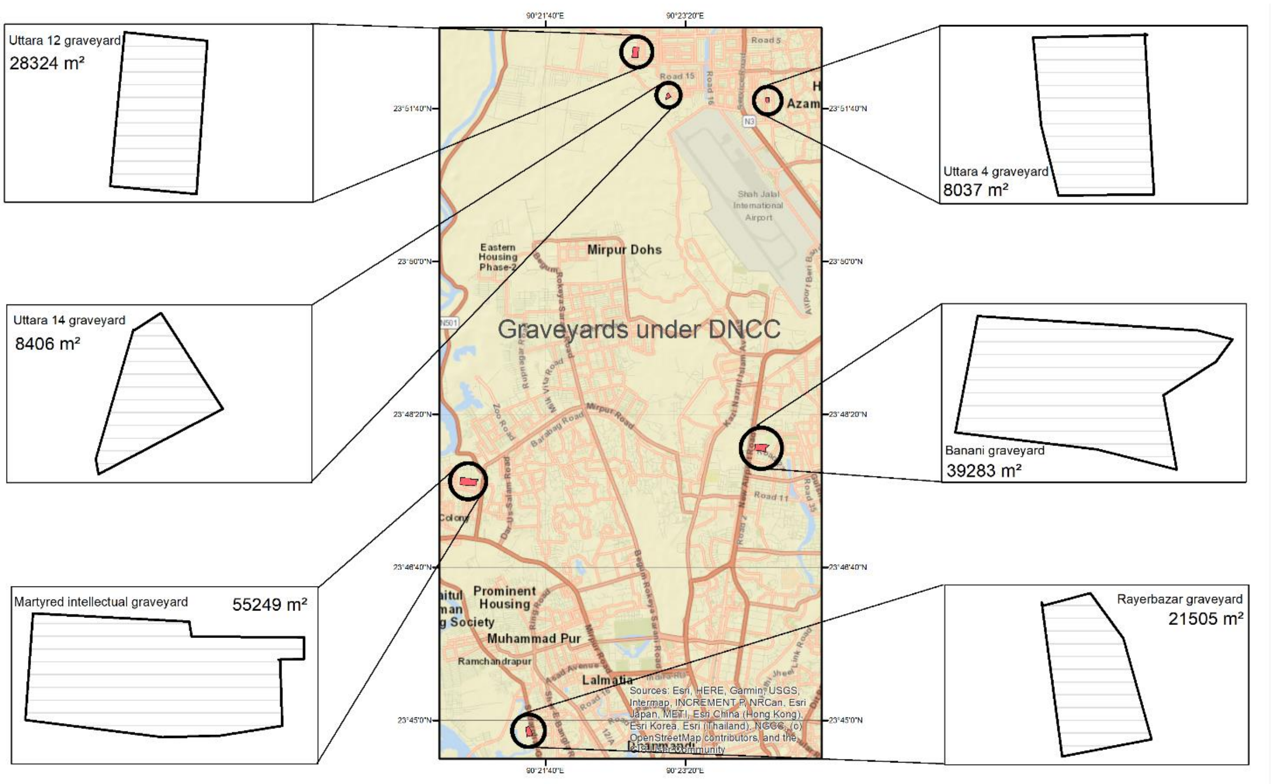

# APPENDIX2: OBSERVATIONAL STRUCTURED TOOL AND CHECKLIST FOR GRAVEYARD DOCUMENTATION PROCESS

**Section 1: General Information**

- **Graveyard Name:**
- **Location:**
- **Date of Observation:**
- **Observer Name:**
- **Time of Observation:**

**Section 2: Graveyard Layout and Environment**

- **Physical Layout:**
    - Clearly marked sections and plots
    - Presence of signage and maps
    - Accessibility (paths, ramps, etc.)

**Section 3: Documentation Practices**

- **Personnel Involved:**
    - Graveyard staff
    - Imam of mosque
    - Other staff
- **Processes Observed:**
    - Recording new burials
    - Updating existing records
    - Maintenance of physical records
    - Digital data entry and updates
- **Frequency:**
    - Daily
    - Weekly
    - Monthly
    - As needed

**Section 4: Tools and Technologies**

- **Manual Tools:**
    - Ledgers/notebooks
    - Physical maps (yes/no)
- **Digital Tools (any digital tool)**

**Section 5: Data Types and Record Keeping**

- **Data Collected:**
    - Names and dates (birth, death)
    - Other details
    - Address of the deceased
    - Epitaphs and inscriptions
    - Photographs of graves
    - Condition and location details.
- **Quality and Accuracy:**
    - Completeness of records
    - Accuracy checks in place
    - Regular updates
- **Storage:**

- Storage to keep register book

**Section 6: Workflow and Procedures**

- **Step-by-Step Workflow:**
  - Initial data collection
  - Data verification
  - Record entry (manual/digital)
  - Maintenance and updates
- **Efficiency:**
  - Streamlined workflow
  - Bottlenecks or delays observed
- **Challenges:**
  - Common obstacles faced
  - Resource limitations (staff, funding, technology)

**Section 7: Engagement with Personnel**

- **Conversations:**
  - Feedback on current practices
  - Challenges highlighted by staff
- **Shadowing:**
  - Detailed observations of daily tasks
  - Interaction patterns among personnel

**Section 8: Assessment of Physical Records**

- **Physical Records:**
  - Condition (well-preserved, damaged)
  - Organisation (orderly, disorganized)
  - Completeness (comprehensive, missing data)
- **Digital Records:**
  - Structure (easy to navigate, complex)
  - Accessibility (readily available, restricted access)

**Section 9: Visual Documentation**

- **Photographs Taken:**
  - Tools and equipment used
  - Workspaces and setup
  - Examples of records (with permissions)
  - General condition of the graveyard

# APPENDIX 3: PHOTOGRAPHS OF PAPER-BASED DOCUMENTATION MATERIALS AND SUPPORTING DOCUMENTS

## Paper-based documentation materials at graveyard

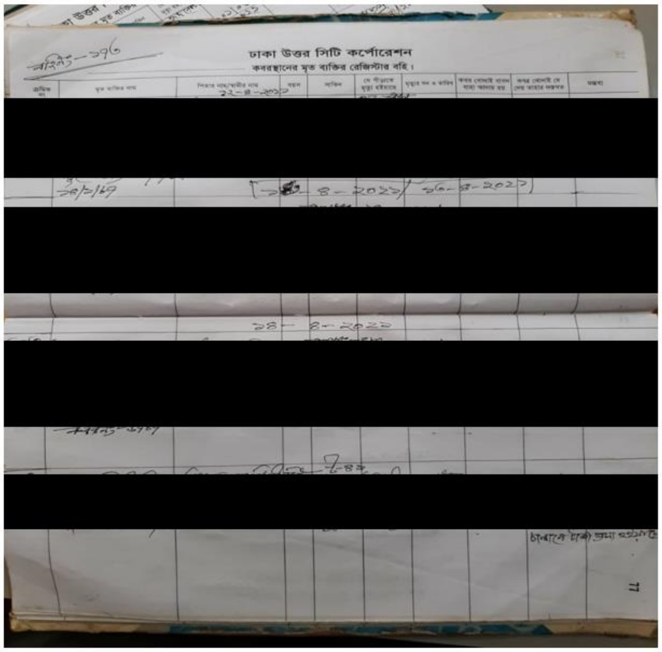
ঢাকা উত্তর সিটি কর্পোরেশন
কবরস্থানের মৃত ব্যক্তির রেজিস্টার বহি।
[redacted]
[redacted]
[redacted]
[redacted]

Register book

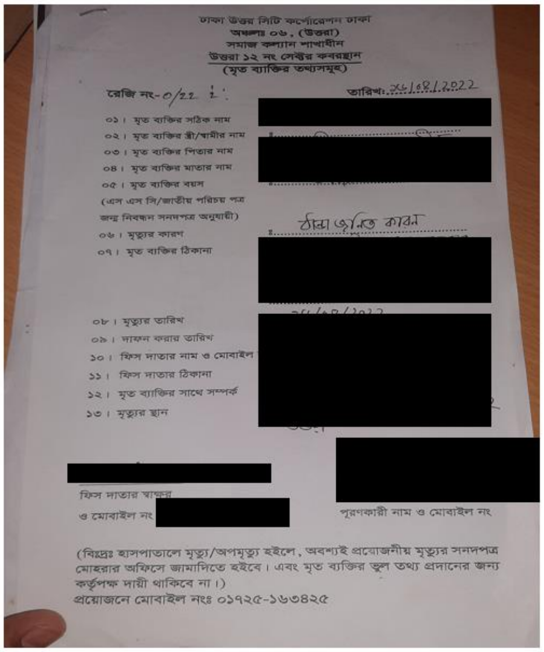
ঢাকা উত্তর সিটি কর্পোরেশন ঢাকা
অঞ্চলঃ ০৬, (উত্তরা)
সমাজ কল্যান শাখাধীন
উত্তরা ১২ নং সেক্টর কবরস্থান
(মৃত ব্যক্তির তথ্যসমূহ)

রেজি নং- 0/22 তারিখঃ 26/08/2022

০১। মৃত ব্যক্তির সঠিক নাম [redacted]
০২। মৃত ব্যক্তির স্ত্রী/স্বামীর নাম
০৩। মৃত ব্যক্তির পিতার নাম [redacted]
০৪। মৃত ব্যক্তির মাতার নাম
০৫। মৃত ব্যক্তির বয়স
(এস এস সি/জাতীয় পরিচয় পত্র জন্ম নিবন্ধন সনদপত্র অনুযায়ী)
০৬। মৃত্যুর কারণ ঠান্ডা জনিত কারন
০৭। মৃত ব্যক্তির ঠিকানা [redacted]
০৮। মৃত্যুর তারিখ [redacted]
০৯। দাফন করার তারিখ
১০। ফিস দাতার নাম ও মোবাইল
১১। ফিস দাতার ঠিকানা
১২। মৃত ব্যক্তির সাথে সম্পর্ক
১৩। মৃত্যুর স্থান

[redacted] [redacted]
ফিস দাতার স্বাক্ষর
ও মোবাইল নং [redacted]
পূরণকারী নাম ও মোবাইল নং

(বিঃদ্রঃ হাসপাতালে মৃত্যু/অপমৃত্যু হইলে, অবশ্যই প্রয়োজনীয় মৃত্যুর সনদপত্র মোহরার অফিসে জমাদিতে হইবে। এবং মৃত ব্যক্তির ভুল তথ্য প্রদানের জন্য কর্তৃপক্ষ দায়ী থাকিবে না।)
প্রয়োজনে মোবাইল নংঃ ০১৭২৫-১৬৩৪২৫

Application form for burial

## Photographs of supporting documents required from relatives of the deceased

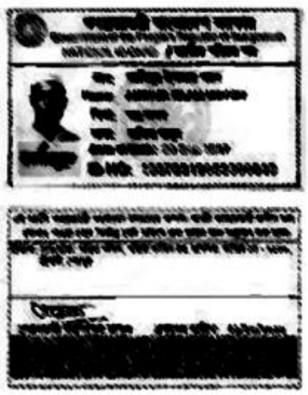

National Identification card

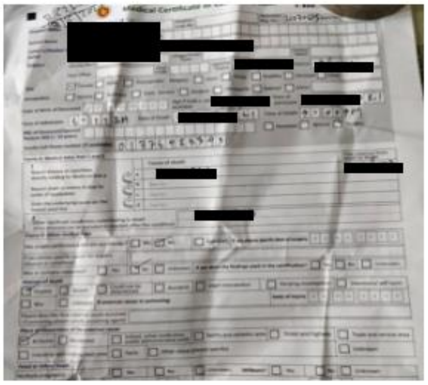

Death certificate

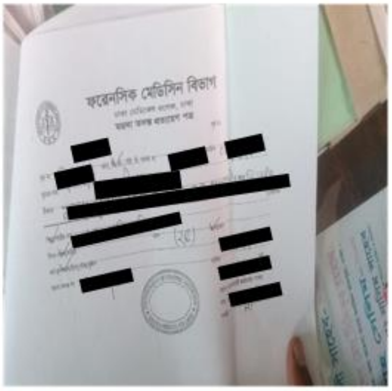

Post mortem report from police

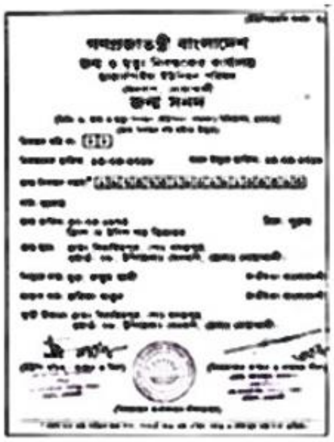

Birth certificate

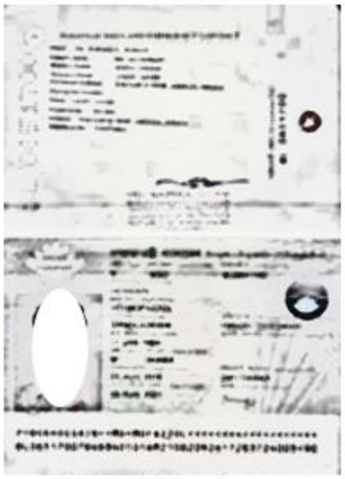

Passport

## Images 1-13 provides the sample copies of the documents we identified in the graveyards.

**Item 1: Draft Register (Khoshra khata)**

This is a special register that was found only in the Banani graveyard (image 1). They use it to draft the information regarding death before recording this information in another register from which they develop reports for City Corporation. In this draft, the caretaker writes the name of the deceased person, father or husbands' name, age, address of the deceased, verifying from the supporting documents such as national ID or death certificates in an unstructured manner.

**Image 1: Draft Register (Khoshra Khata)**

চিঠিপত্র গ্রহণের রেজিস্টার

ঢাকা সিটি করপোরেশন



| [illegible] | [illegible] | [illegible] | [illegible] |
|---|---|---|---|
| [redacted] | [redacted] | | |
| বয়স - 74 বছর, [illegible] | মোবা: [redacted] | | |
| মৃত্যু তাং - 30.04.2021 | পিতা: [redacted] | | |
| মৃত্যু কারণ - | বয়স: [redacted] | | |
| [redacted] | ঠিকানা: [redacted] | | |
| বয়স - 74 বছর | মৃত্যুর সময় ও তারিখ - 02-02-22 | | |
| [redacted] -2022 | মৃত্যুর কারণ - [illegible] | | |
| [redacted] | মৃত্যুর স্থান: Labaid Hospital | | |
| মৃত্যু কারণ - নিজ বাসায় | ফোন- [redacted] | | |
| [redacted] | পেশা: [redacted] | | |

**Item 2: Form for death information**

The graveyards in Uttara (sectors 4, 12, and 14) use a structured form for collecting the initial information of the deceased person before burial. The initial information includes the name of the deceased, mother, father and husband or wife's name, age, address, place and cause of death, contact number of the relative and the relation between the deceased and information provider.

**Image 2: Form to collect information about deceased**

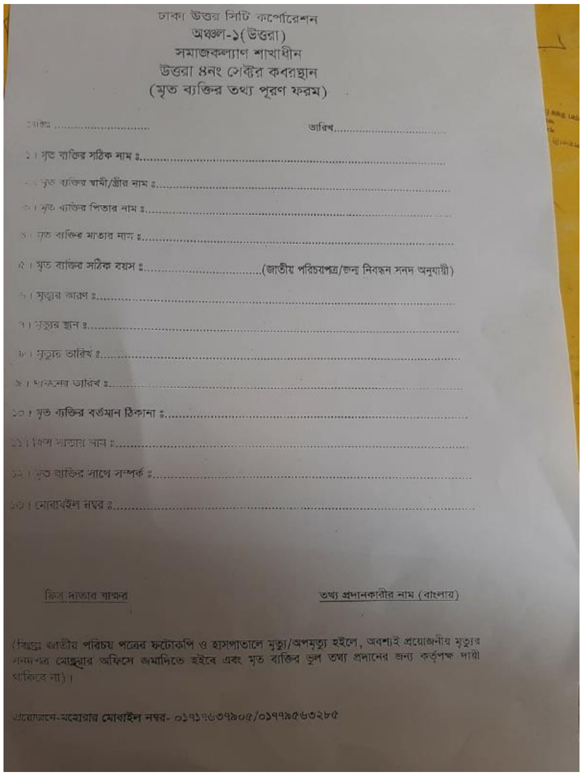

ঢাকা উত্তর সিটি কর্পোরেশন
অঞ্চল-১(উত্তরা)
সমাজকল্যাণ শাখাধীন
উত্তরা ৪নং সেক্টর কবরস্থান
(মৃত ব্যক্তির তথ্য পূরণ ফরম)

[illegible]........................ তারিখ........................

১। মৃত ব্যক্তির সঠিক নাম ঃ..........................................

২। মৃত ব্যক্তির স্বামী/স্ত্রীর নাম ঃ..........................................

৩। মৃত ব্যক্তির পিতার নাম ঃ..........................................

৪। মৃত ব্যক্তির মাতার নাম ঃ..........................................

৫। মৃত ব্যক্তির সঠিক বয়স ঃ........................(জাতীয় পরিচয়পত্র/জন্ম নিবন্ধন সনদ অনুযায়ী)

৬। মৃত্যুর কারণ ঃ..........................................

৭। মৃত্যুর স্থান ঃ..........................................

৮। মৃত্যুর তারিখ ঃ..........................................

৯। দাফনের তারিখ ঃ..........................................

১০। মৃত ব্যক্তির বর্তমান ঠিকানা ঃ..........................................

১১। তথ্য দাতার নাম ঃ..........................................

১২। মৃত ব্যক্তির সাথে সম্পর্ক ঃ..........................................

১৩। মোবাইল নম্বর ঃ..........................................

তথ্য দাতার স্বাক্ষর — তথ্য প্রদানকারীর নাম (বাংলায়)

(বিঃদ্রঃ জাতীয় পরিচয় পত্রের ফটোকপি ও হাসপাতালে মৃত্যু/অপমৃত্যু হইলে, অবশ্যই প্রয়োজনীয় মৃত্যুর সনদপত্র মোহুরার অফিসে জমাদিতে হইবে এবং মৃত ব্যক্তির ভুল তথ্য প্রদানের জন্য কর্তৃপক্ষ দায়ী থাকিবে না)।

প্রয়োজনে-মহোরার মোবাইল নম্বর- ০১৭১৭৬৩৭৯০৫/০১৭৭৯৫৬৩২৮৫

**Item 3: Post-mortem report**

When a person dies due to unnatural causes, the caretaker collects a copy of the post-mortem report (image 3). This practice is not uniform across all graveyards.

**Image 3: Post-mortem report certification**

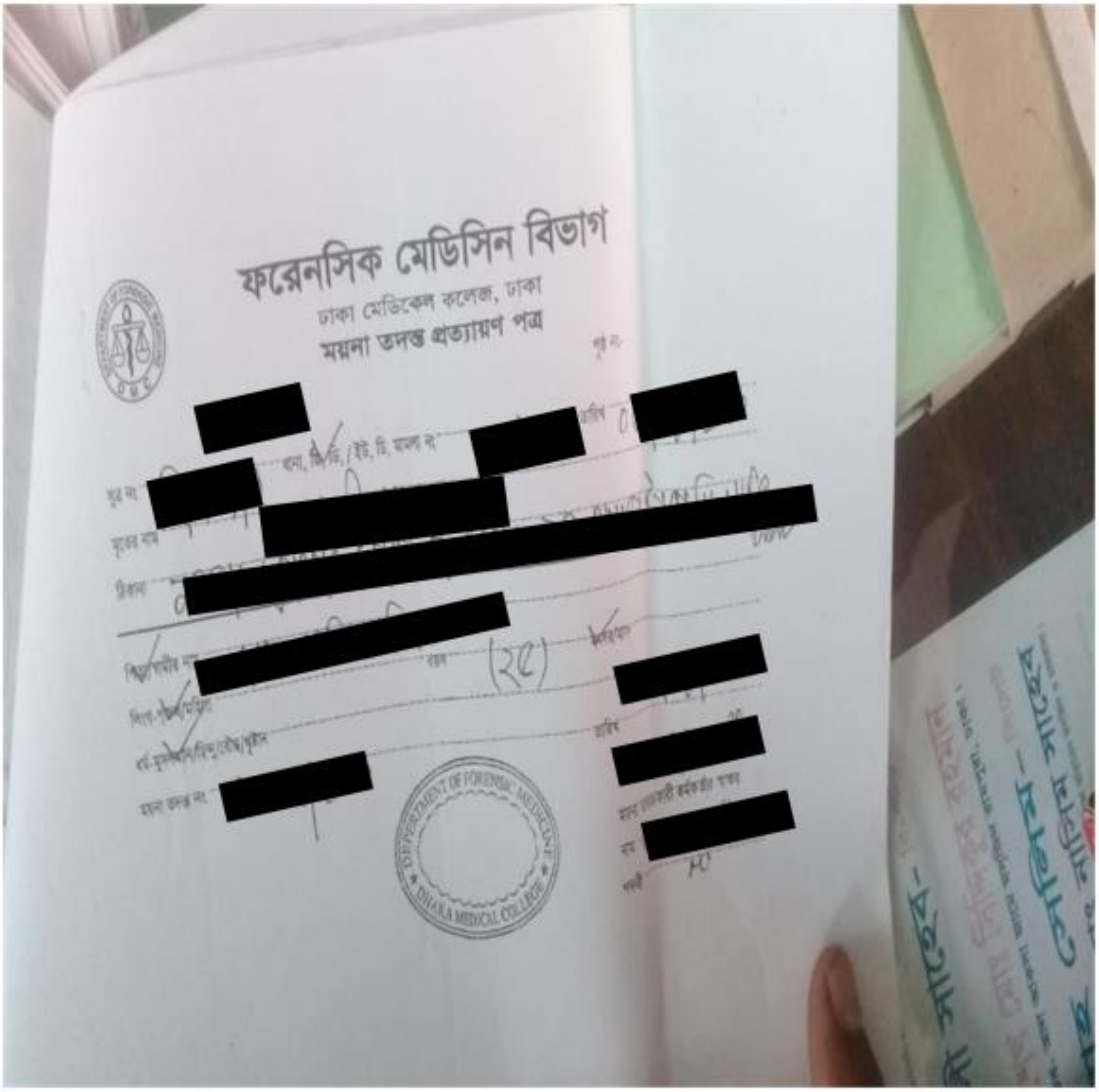

**Item 4: Medical certificate**

A medical certificate is collected as a supporting document if the death has happened in the hospital (Image 4). This practice is not uniform across all graveyards.

**Image 4: Medical certificate for death**

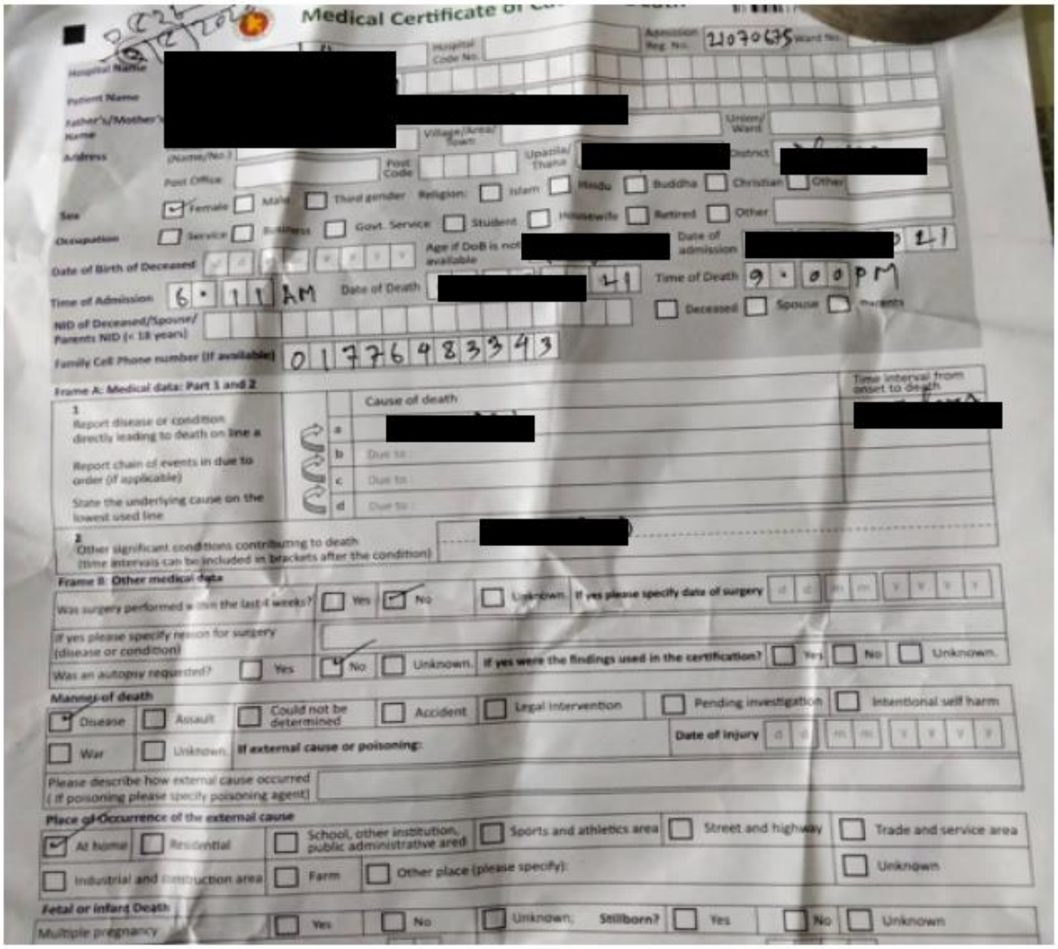

Medical Certificate of [illegible]

Hospital Name [redacted] Hospital Code No. Admission Reg. No. 21070675 Ward No.

Patient Name

Father's/Mother's Name

Address (Name/No.) Village/Area/Town Union/Ward

Post Office Post Code Upazila/Thana [redacted] District [redacted]

Sex: ☑ Female ☐ Male ☐ Third gender Religion: ☐ Islam ☐ Hindu ☐ Buddha ☐ Christian ☐ Other

Occupation: ☐ Service ☐ Business ☐ Govt. Service ☐ Student ☐ Housewife ☐ Retired ☐ Other

Date of Birth of Deceased Age if DoB is not available [redacted] Date of admission [redacted] 21

Time of Admission 6 • 11 AM Date of Death [redacted] 21 Time of Death 9 • 00 PM

NID of Deceased/Spouse/Parents NID (< 18 years) ☐ Deceased ☐ Spouse ☐ Parents

Family Cell Phone number (if available) 01776483343

Frame A: Medical data: Part 1 and 2

| | | Cause of death | Time interval from onset to death |
|---|---|---|---|
| 1 Report disease or condition directly leading to death on line a | a | [redacted] | [redacted] |
| Report chain of events in due to order (if applicable) | b | Due to: | |
| | c | Due to: | |
| State the underlying cause on the lowest used line | d | Due to: | |
| 2 Other significant conditions contributing to death (time intervals can be included in brackets after the condition) | | [redacted] | |

Frame B: Other medical data

Was surgery performed within the last 4 weeks? ☐ Yes ☑ No ☐ Unknown. If yes please specify date of surgery d d m m y y y y

If yes please specify reason for surgery (disease or condition)

Was an autopsy requested? ☐ Yes ☑ No ☐ Unknown. If yes were the findings used in the certification? ☐ Yes ☐ No ☐ Unknown.

Manner of death

☑ Disease ☐ Assault ☐ Could not be determined ☐ Accident ☐ Legal intervention ☐ Pending investigation ☐ Intentional self harm

☐ War ☐ Unknown. If external cause or poisoning: Date of injury d d m m y y y y

Please describe how external cause occurred (If poisoning please specify poisoning agent)

Place of Occurrence of the external cause

☑ At home ☐ Residential ☐ School, other institution, public administrative area ☐ Sports and athletics area ☐ Street and highway ☐ Trade and service area

☐ Industrial and construction area ☐ Farm ☐ Other place (please specify): ☐ Unknown

Fetal or infant Death

Multiple pregnancy ☐ Yes ☐ No ☐ Unknown; Stillborn? ☐ Yes ☐ No ☐ Unknown

**Item 5: Death certificate**

A death certificate undersigned by the city corporation counsellor is collected if the death has happened at home (image: To be collected). This practice is not uniform across all graveyards and within the same graveyard.

**Image 5: Death certificate**

## নকলের জন্য পাতা
## Folio For Copies

রায়ের বাজার কবরস্থানের মৃত ব্যক্তির রেজিষ্টার বহি

| ক্রমিক নং | মৃত ব্যক্তির নাম | পিতা/স্বামীর নাম | বয়স | সাকিন | যে পীড়াতে মৃত্যু হইয়াছে | মৃত্যুরসন ও তারিখ | কবর খোদাই বাবদ যাহা আদায় হয় | কবর খোদাই যে দেয় তাহার দস্তখত | মন্তব্য |
|---|---|---|---|---|---|---|---|---|---|
| [redacted] | [redacted] | [redacted] | ৬১ বৎসর | [redacted] | CD-১৯ করোনা<br>বি,এস,এম,এম,ইউ, ঢাকা। | ১৫/০৭ ২০২০ | ফ্রি | [redacted] | |

[redacted]
(Copies by)

[redacted]
(Compared by)

সত্য বলিয়া প্রমানিত নকল
Certified as Correct Copies

[redacted]
সাধারণ প্রশাসন শাখা
ঢাকা উত্তর সিটি কর্পোরেশন

**Item 6: National ID**

A copy of the national ID card is collected in all graveyards. The Rayerbazar graveyard do not practice this consistently.

**Image 6: National ID care**

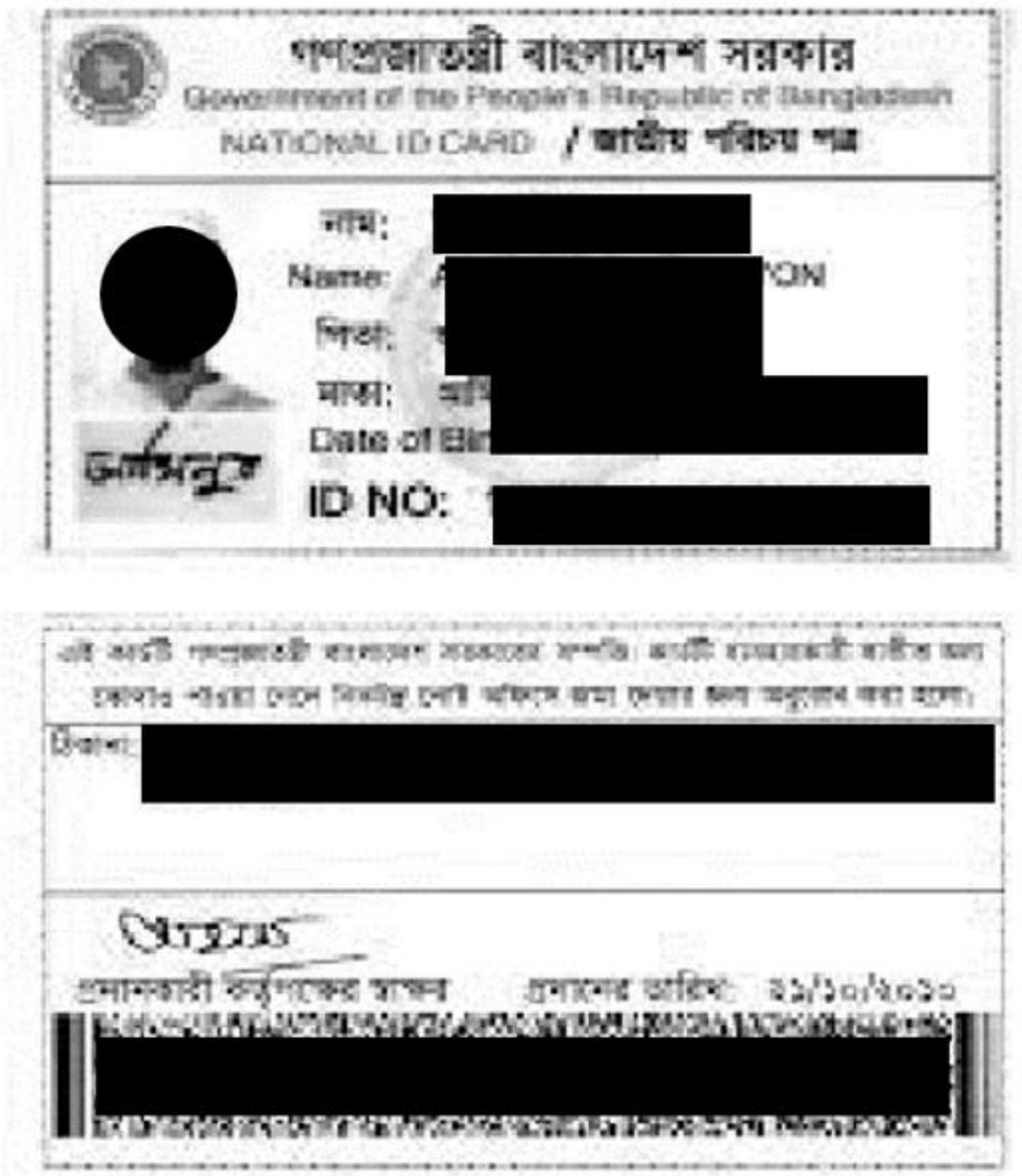

গণপ্রজাতন্ত্রী বাংলাদেশ সরকার
Government of the People's Republic of Bangladesh
NATIONAL ID CARD / জাতীয় পরিচয় পত্র

নাম: [redacted]
Name: [redacted]
পিতা: [redacted]
মাতা: [redacted]
Date of Bi[redacted]
ID NO: [redacted]

এই কার্ডটি গণপ্রজাতন্ত্রী বাংলাদেশ সরকারের সম্পত্তি। কার্ডটি ব্যবহারকারী ব্যতীত অন্য কোথাও পাওয়া গেলে নিকটস্থ পোস্ট অফিসে জমা দেবার জন্য অনুরোধ করা হলো।

ঠিকানা: [redacted]

প্রদানকারী কর্তৃপক্ষের স্বাক্ষর    প্রদানের তারিখ: [illegible]

[redacted]

**Item 7: Death Register: General**

In general, there are two types of registers in each graveyard. One structured register is dedicated to general graves (image 7). The general register includes the following variables: variables of interest are name, father or husband's name, age, address, cause of death, date of death, the amount of money paid for the burial, signature of the authorized person and remarks. The practice is reasonably consistent across all graveyards. The Mirpur and Uttara Sector 4 graveyards collect information regarding the place of death under the "remarks" column. However, other graveyards do not document this information in the registers.

**Image 7: Death register for general graves**

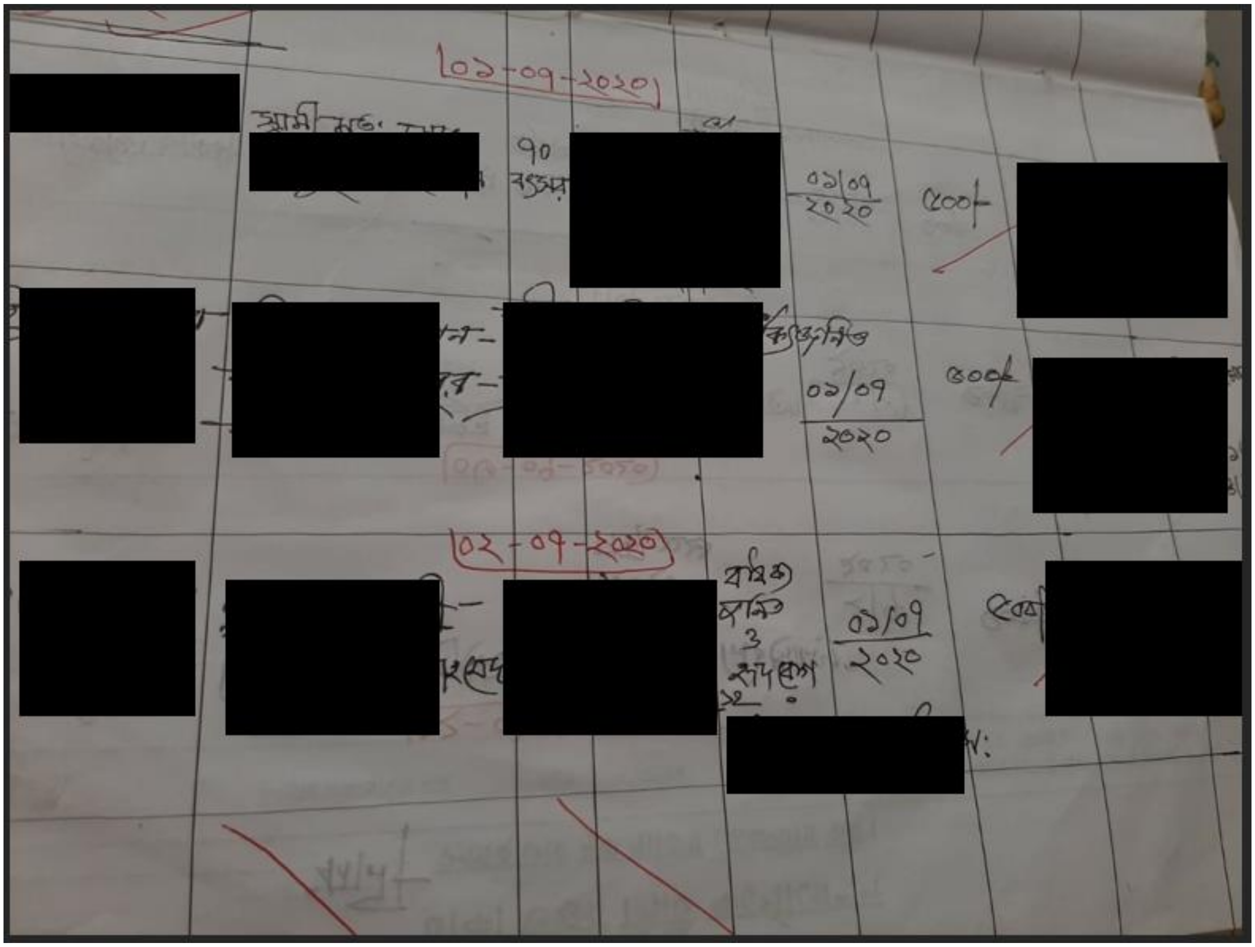

**Item 8: Death Register for reserved graves**

The second type of register is dedicated to the reserved graves. A grave can be reserved for a family for 15 or 25 years based on special one-time payment. The following variables are included in the register: name of the deceased person, father or husband's name, age, address, cause of death, date of death, amount of money paid for the burial, the signature of the authorized person and remarks. This practice is not uniform across all graveyards. The Uttara 12 graveyard maintain a hand written register for reserved graves. Also, they record the death information of reserved graves in the general register. There is no unique ID to match the entries. Hence the only way to match these records is by comparing the deceased names and other personal details

Each reserved grave has a unique identification number. However, general graves do not have any identification numbers.

**Image 7: Death Register for reserved graves**

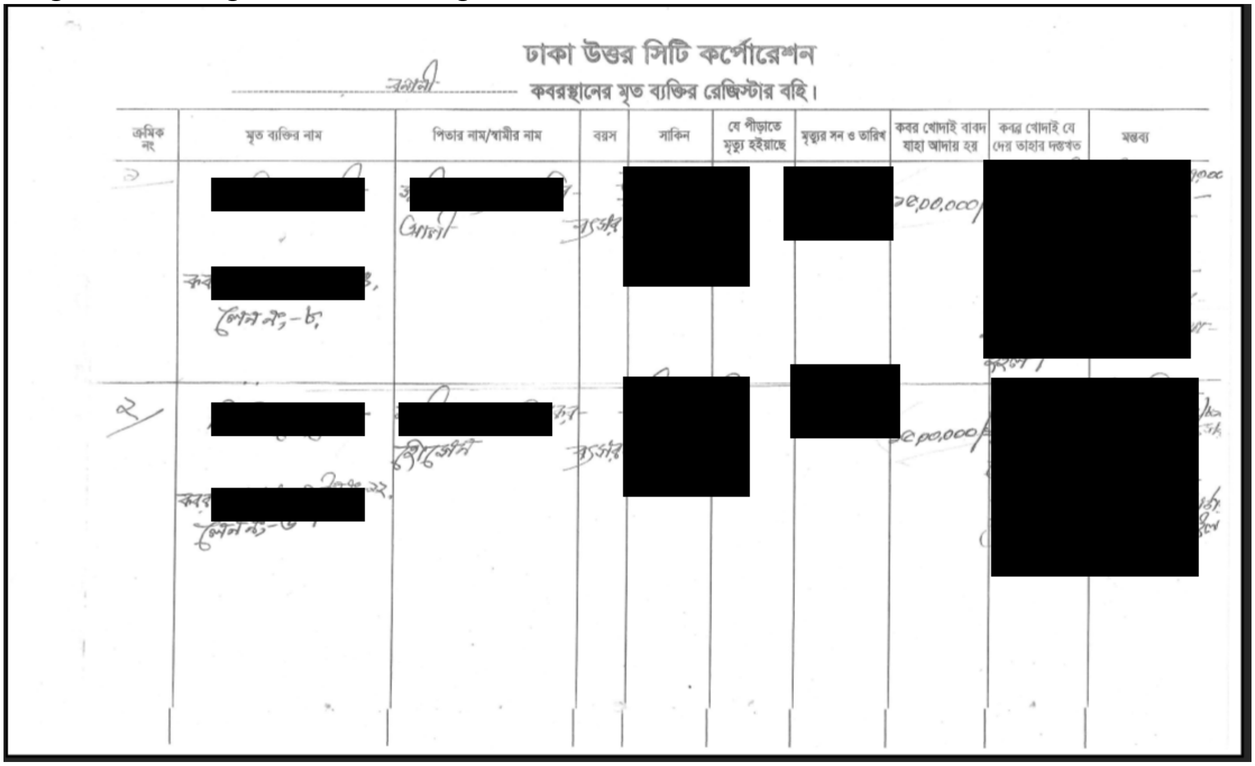

ঢাকা উত্তর সিটি কর্পোরেশন

[illegible] কবরস্থানের মৃত ব্যক্তির রেজিস্টার বহি।

| ক্রমিক নং | মৃত ব্যক্তির নাম | পিতার নাম/স্বামীর নাম | বয়স | সাকিন | যে পীড়াতে মৃত্যু হইয়াছে | মৃত্যুর সন ও তারিখ | কবর খোদাই বাবদ যাহা আদায় হয় | কবর খোদাই যে দেয় তাহার দস্তখত | মন্তব্য |
|---|---|---|---|---|---|---|---|---|---|
| ১ | [redacted] [redacted] [illegible] | [redacted] [illegible] | [illegible] | [redacted] | | [redacted] | [illegible] | [redacted] | [illegible] |
| ২ | [redacted] [redacted] [illegible] | [redacted] [illegible] | [illegible] | [redacted] | | [redacted] | [illegible] | [redacted] | [illegible] |

**Item 9: Death Register: Reuse of the reserved graves**

The Uttara Sector 4 graveyard uses another dedicated register for reusing the reserved graveyards for immediate family members. This register has similar variables as the general register. However, the financial mechanism is different for reusing a reserved grave.

**Image 8: Death Register for reuse**

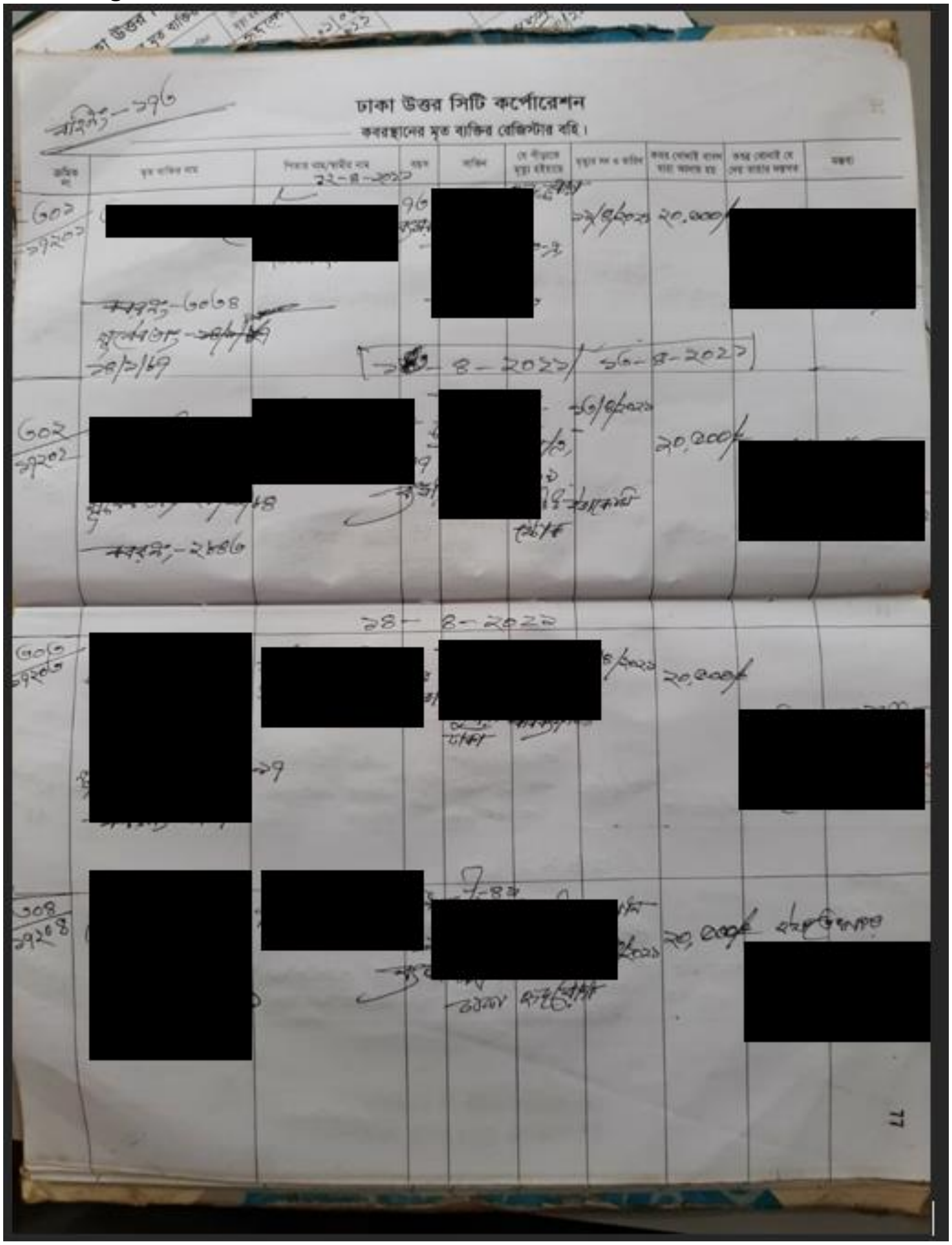

**Item 10: Money receipt**

The graveyards collets a processing charge for each burial. The rates are different for general and reserved graves. The caretaker issues a money receipt to document the financial transaction. The following information is documented in the money receipt: name of the deceased person, father's name, age, address, size of the grave, and name and address of the information provider.

**Image 10: Money receipt for graves**

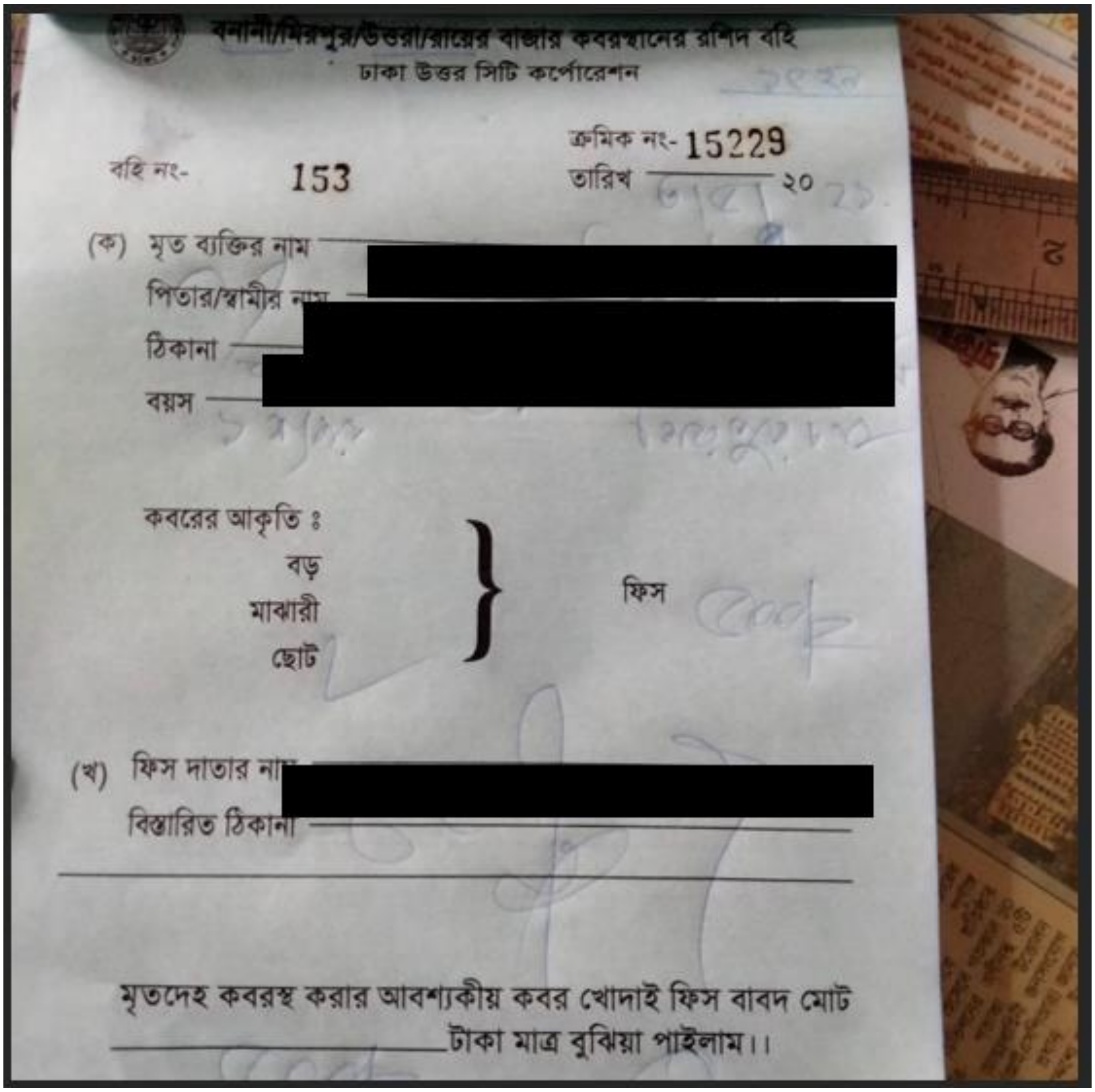

বনানী/মিরপুর/উত্তরা/রায়ের বাজার কবরস্থানের রশিদ বহি
ঢাকা উত্তর সিটি কর্পোরেশন

ক্রমিক নং- 15229

বহি নং- 153

তারিখ ______ ২০

(ক) মৃত ব্যক্তির নাম [redacted]
পিতার/স্বামীর না[redacted]
ঠিকানা [redacted]
বয়স [redacted]

কবরের আকৃতি ঃ
বড়
মাঝারী
ছোট
} ফিস

(খ) ফিস দাতার না[redacted]
বিস্তারিত ঠিকানা ______

মৃতদেহ কবরস্থ করার আবশ্যকীয় কবর খোদাই ফিস বাবদ মোট ______ টাকা মাত্র বুঝিয়া পাইলাম।।

**Item 11: Challan (Invoice)**

The caretaker deposits the money collected as burial processing charges to the Mayor's Bank Account through an invoice (challan) (image 11). However, the number of copies of challan was different across different graveyards. The following information is available in the challan: serial number of the death in invoice, date and serial number of the register, name and address of the deceased, contact details of one of the relatives of the deceased, area of the grave (reserved or general), fees paid, receipt number and date and remarks. The caretaker keeps a copy of the invoice in the archive for audit purpose. The practice is consistent across all graveyards.

**Image 11: Challan Copy**

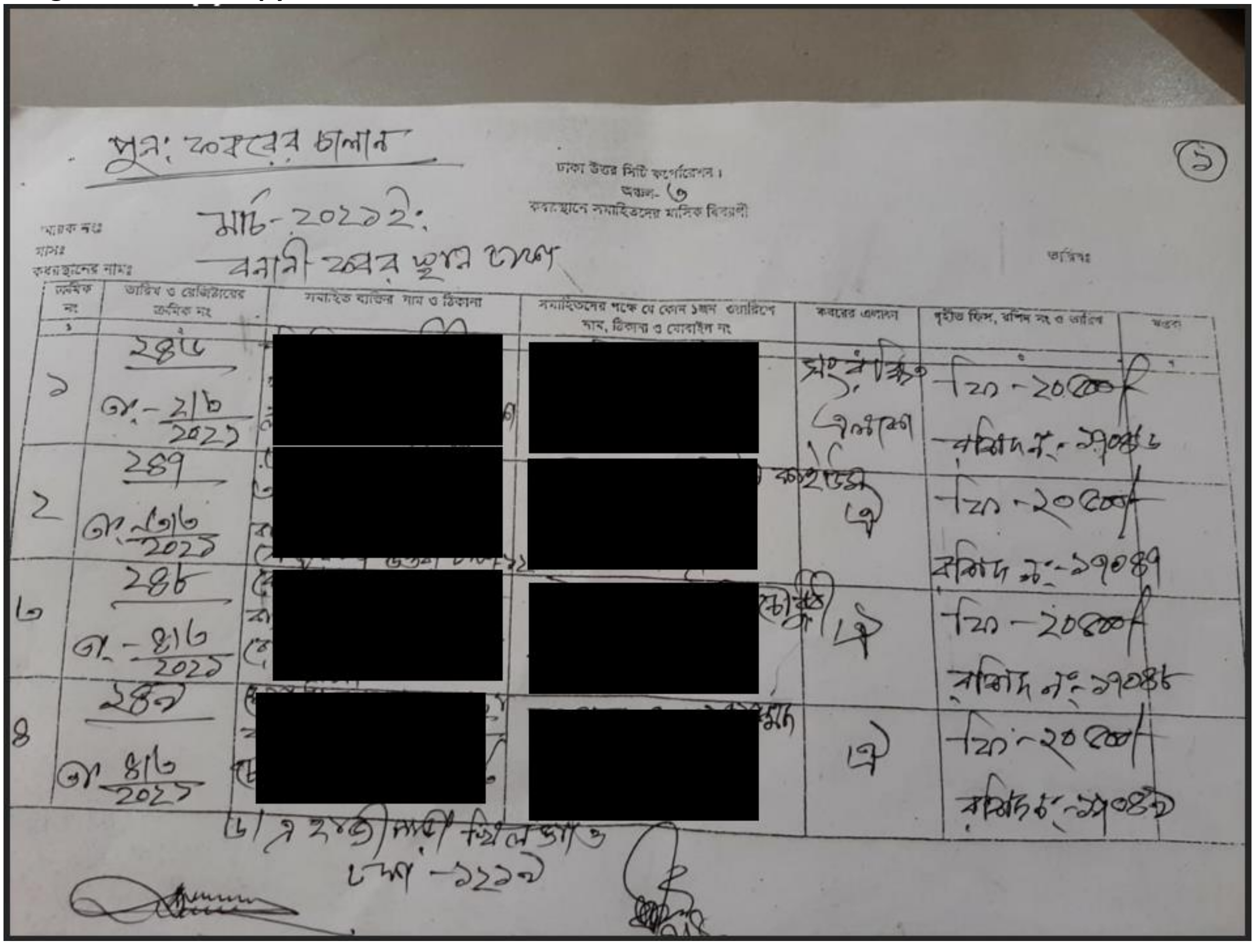

পুনঃ কবরের চালান

ঢাকা উত্তর সিটি কর্পোরেশন।
অঞ্চল- ৩
কবরস্থানে সমাহিতদের মাসিক বিবরণী

স্মারক নংঃ
নামঃ
কবরস্থানের নামঃ
তারিখঃ

| ক্রমিক নং | তারিখ ও রেজিস্টারের ক্রমিক নং | সমাহিত ব্যক্তির নাম ও ঠিকানা | সমাহিতদের পক্ষে যে কোন ১জন ওয়ারিশের নাম, ঠিকানা ও মোবাইল নং | কবরের এলাকা | গৃহীত ফিস, রশিদ নং ও তারিখ | মন্তব্য |
|---|---|---|---|---|---|---|
| ১ | [illegible] | [redacted] | [redacted] | [illegible] | ফি - ২০০০/- রশিদ নং - [illegible] | |
| ২ | [illegible] | [redacted] | [redacted] | [illegible] | ফি - ২০০০/- রশিদ নং - [illegible] | |
| ৩ | [illegible] | [redacted] | [redacted] | [illegible] | ফি - ২০০০/- রশিদ নং - [illegible] | |
| ৪ | [illegible] | [redacted] | [redacted] | [illegible] | ফি - ২০০০/- রশিদ নং - [illegible] | |

**Item 12: Weekly report to Health Division of City Corporation**

Each week the caretaker sends a structured report to the Health Division of the City Corporation office (image 12). The following information is available in the report: date of registration of the death, place of death, date of death, name of deceased and address, sex, age (year, month, day), nationality and religion, cause of death, occupation, particulars of father or husband and religion and remarks. Although the caretakers are supposed to send the report every week, they send the report monthly in practice. The practice is reasonably consistent across all graveyards.

**Image 12: Weekly death report to Health division**

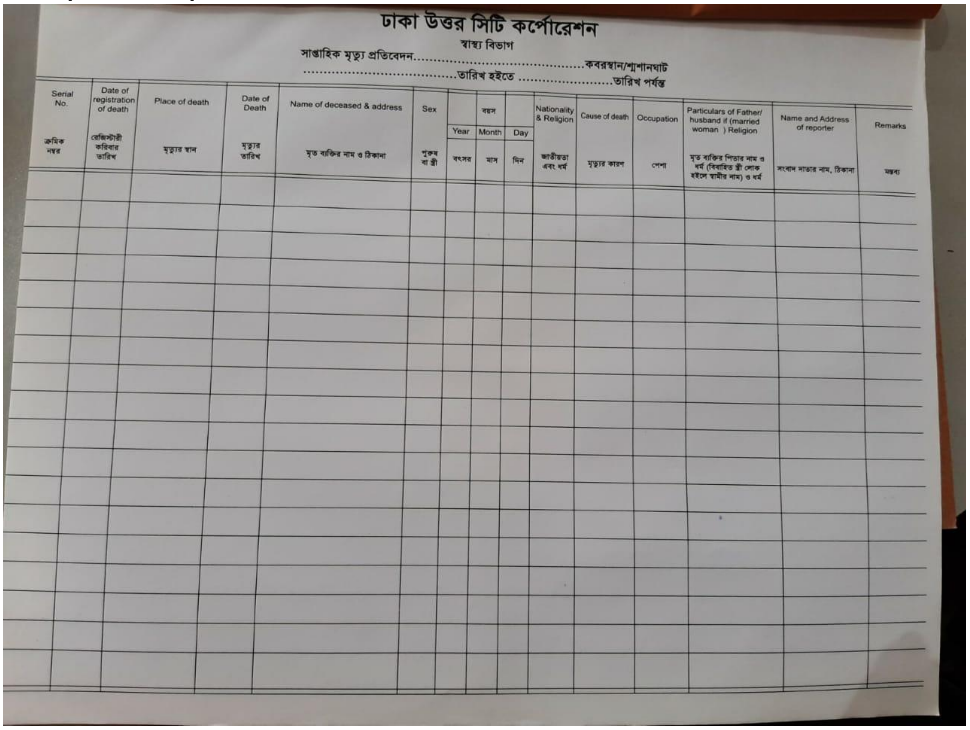

ঢাকা উত্তর সিটি কর্পোরেশন

স্বাস্থ্য বিভাগ

সাপ্তাহিক মৃত্যু প্রতিবেদন........................................কবরস্থান/শ্মশানঘাট

.....................................তারিখ হইতে ........................তারিখ পর্যন্ত

| Serial No. | Date of registration of death | Place of death | Date of Death | Name of deceased & address | Sex | বয়স | | | Nationality & Religion | Cause of death | Occupation | Particulars of Father/ husband if (married woman ) Religion | Name and Address of reporter | Remarks |
|---|---|---|---|---|---|---|---|---|---|---|---|---|---|---|
| | | | | | | Year | Month | Day | | | | | | |
| ক্রমিক নম্বর | রেজিস্টারী করিবার তারিখ | মৃত্যুর স্থান | মৃত্যুর তারিখ | মৃত ব্যক্তির নাম ও ঠিকানা | পুরুষ বা স্ত্রী | বৎসর | মাস | দিন | জাতীয়তা এবং ধর্ম | মৃত্যুর কারণ | পেশা | মৃত ব্যক্তির পিতার নাম ও ধর্ম (বিবাহিত স্ত্রী লোক হইলে স্বামীর নাম) ও ধর্ম | সংবাদ দাতার নাম, ঠিকানা | মন্তব্য |

**Item 13: Monthly income report to Social Welfare of City Corporation**

A monthly report is sent to the social welfare division of the City Corporation Office with the income information each month from each graveyard (image 13). The following information is available in the report: description of income, number of graves, amount of money received and remarks. The practice is reasonably consistent across all graveyards. However, there are slight variations in the monthly income report template across different graveyards.

**Image 13: Monthly income report to Social Welfare Division of City Corporation**

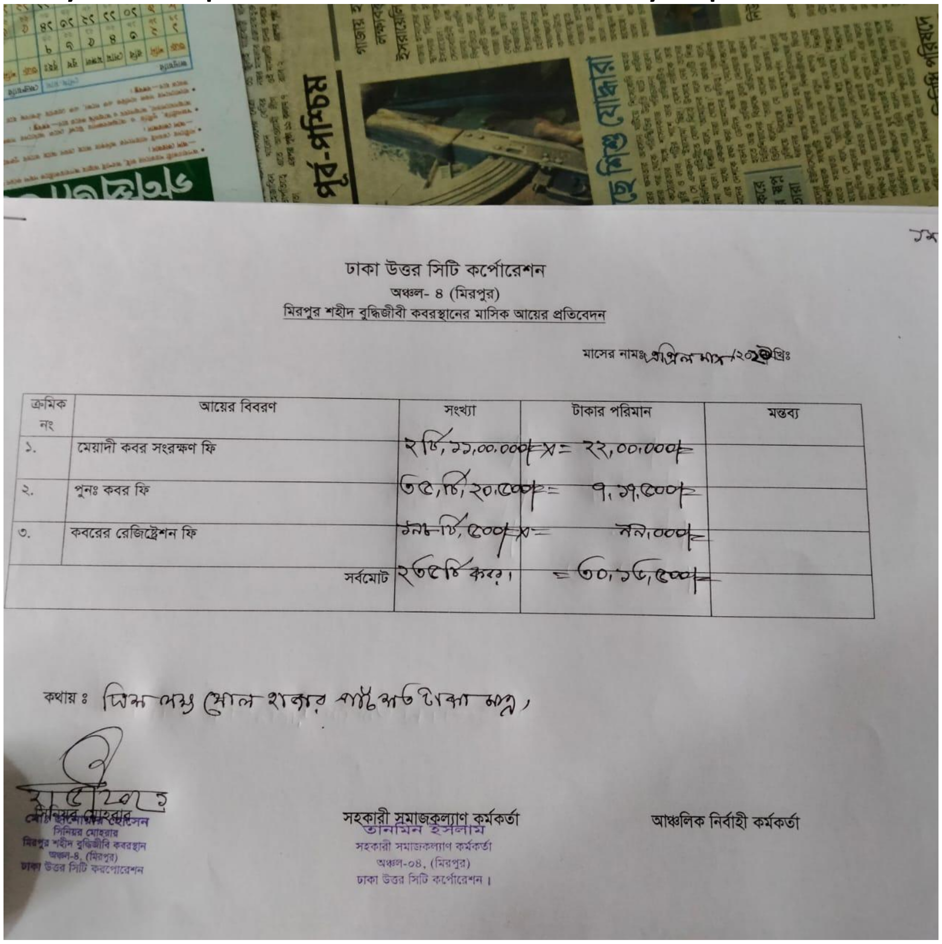

ঢাকা উত্তর সিটি কর্পোরেশন
অঞ্চল- ৪ (মিরপুর)
মিরপুর শহীদ বুদ্ধিজীবী কবরস্থানের মাসিক আয়ের প্রতিবেদন

মাসের নামঃ এপ্রিল মাস/২০২[illegible] খ্রিঃ

| ক্রমিক নং | আয়ের বিবরণ | সংখ্যা | টাকার পরিমান | মন্তব্য |
|---|---|---|---|---|
| ১. | মেয়াদী কবর সংরক্ষণ ফি | ২টি, ১১,০০,০০০/- x | = ২২,০০,০০০/- | |
| ২. | পুনঃ কবর ফি | ৩৫,টি, ২০,৫০০/- | = ৭,১৭,৫০০/- | |
| ৩. | কবরের রেজিষ্ট্রেশন ফি | ১৯৮টি, ৫০০/- x | = ৯৯,০০০/- | |
| | সর্বমোট | ২৩৫টি কবর। | = ৩০,১৬,৫০০/- | |

কথায়ঃ ত্রিশ লক্ষ ষোল হাজার পাঁচশত টাকা মাত্র।

সিনিয়র মোহরার
মোঃ [illegible] হোসেন
সিনিয়র মোহরার
মিরপুর শহীদ বুদ্ধিজীবি কবরস্থান
অঞ্চল-৪, (মিরপুর)
ঢাকা উত্তর সিটি করপোরেশন

সহকারী সমাজকল্যাণ কর্মকর্তা
তানমিন ইসলাম
সহকারী সমাজকল্যাণ কর্মকর্তা
অঞ্চল-০৪, (মিরপুর)
ঢাকা উত্তর সিটি কর্পোরেশন।

আঞ্চলিক নির্বাহী কর্মকর্তা

Table 3 summarises the mapping of the process for graveyard documentation. In summary, the process of documentation can be explained in three broad steps: entering the data in registers, financial processing and, reporting to city corporation regarding these deaths' as well as graveyard income information.

**Table 3: Mapping of the process of documentation in the graveyards**

| Mapping of the process | Items used |
|---|---|
| Entering death data into the registers | 1. Draft Register (Khoshra khata)<br>2. Form for death information<br>3. Post-mortem report<br>4. Medical certificate<br>5. Death Certificate (from CC)<br>6. NID<br>7. Death Register: General<br>8. Death Register: Reservation<br>9. Death Register: Reuse of the reserved graves |
| Financial process | 10. Money receipt<br>11. Challan |
| Reporting the death and income information to City Corporation | 12. Monthly income report to Social Welfare of City Corporation<br>13. Weekly report to Health Division of City Corporation |

# APPENDIX 4: IN-DEPTH INTERVIEW TOOL TO ASSESS THE DOCUMENTATION PROCESS IN GRAVEYARD

**Overview**
Can you briefly describe the current documentation process for burial records in the graveyard?
**Record Keeping**
What are the main challenges you face with maintaining accurate and complete records?
**Accessibility and Security**
How do you ensure that the burial records are both secure and accessible when needed?
**Improvements**
What improvements would you suggest to enhance the documentation process?
**Future Prospects**
Are there any upcoming changes planned for the documentation system? If so, what are they?
**Conclusion**
Is there anything else you would like to add about the documentation process?

# APPENDIX 5: ELECTRONIC GRAVEYARD DATA ENTRY SOFTWARE TO RECORD HISTORICAL DATA FROM GRAVEYARDS

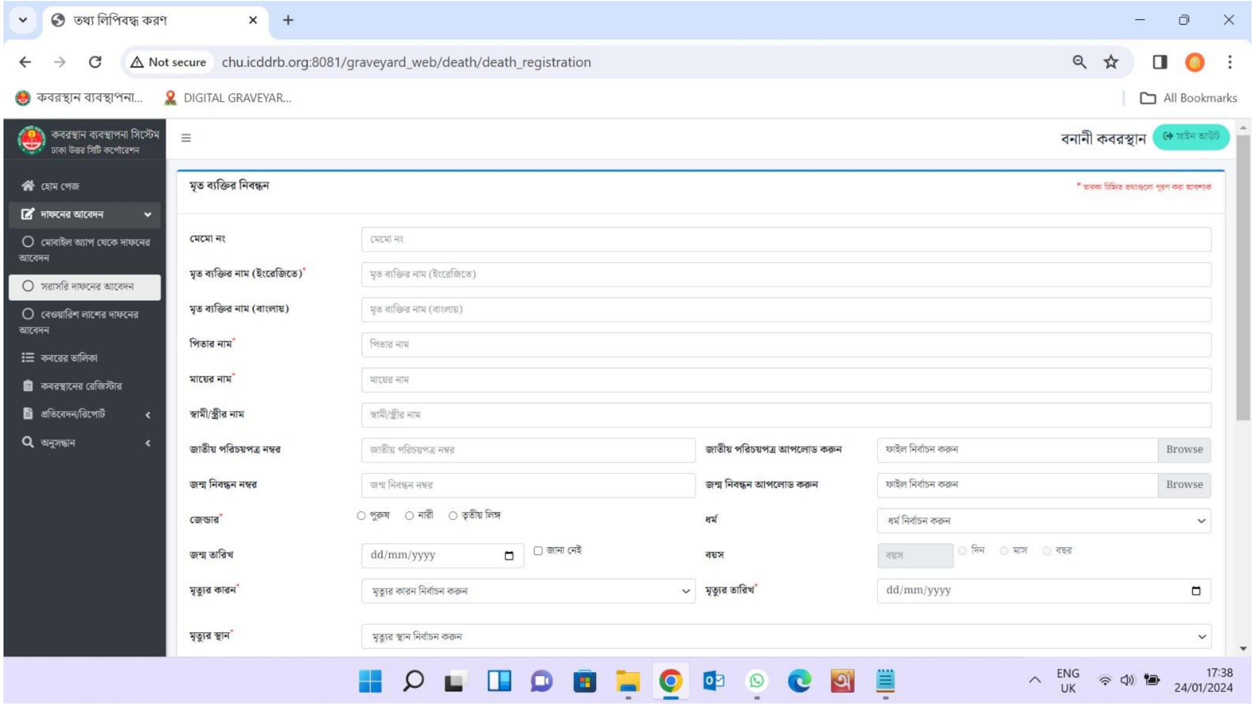

# APPENDIX 6: DEVELOPMENT AND IMPLEMENTATION OF DIGITAL GRAVEYARD DEATH RECORD KEEPING SYSTEM

### Development of digital graveyard death record keeping system

Following the development of the architecture by stakeholder consultations (Figure 2), we developed an integrated system comprising multiple platforms and applications to streamline digital graveyard management. We created a mobile app (Appendix 7a) and website (Appendix 7b) that allowed users to submit applications for grave reservations, access information about the graveyard, and obtain details about funeral procedures. For graveyard supervisors, we designed a death record-keeping platform to efficiently manage and update burial records (Appendix 7c). Additionally, a manager platform was developed for Dhaka North City Corporation (DNCC) managers to view weekly and monthly reports and monitor the status of all graveyards under their jurisdiction. The manager platform also has a dashboard for Dhaka North City Corporation (DNCC) managers, which included a robust dashboard providing trend analysis, distribution data, and weekly, monthly, and yearly projections (Appendix 7d). We created digital maps of six graveyards, providing high-resolution aerial views and accurate layouts by utilising drone technology, and then developed a web application for kiosk systems installed at the graveyards, allowing visitors to search for information about their relatives and navigate to specific graves using the interactive map (Appendix 7e).

### Implementation of digital graveyard death record keeping system

To implement the system, we uploaded the mobile app to the Google Play Store and launched the accompanying website. The Dhaka North City Corporation (DNCC) held a launching program to introduce and promote these platforms. From April 2022 to August 2022, we established computer setups, including tables, chairs, UPS units, printers, paper, ink, and internet connections, in all six graveyards (figure below).

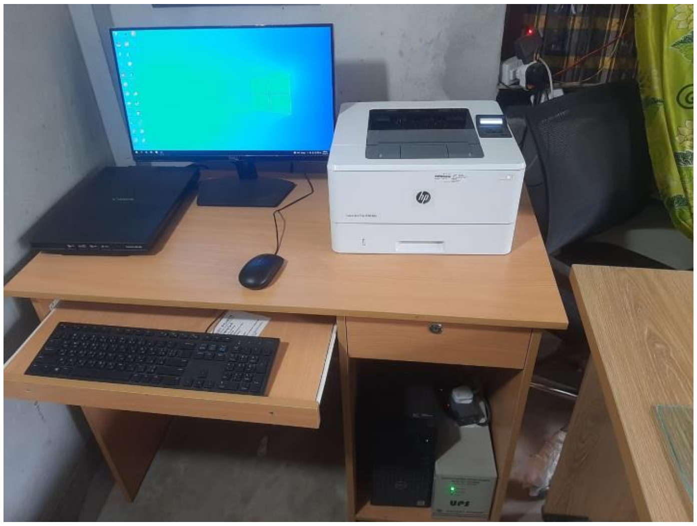

Notably, none of the graveyard managers had prior training on computer use. To address this, two members of the developer team provided training to four graveyard supervisors. This training covered basic computer skills and the use of the digital death record-keeping system. The training sessions were conducted twice a month for six months, with each session lasting two hours. Additionally, the graveyard managers were trained on the digital death record-keeping system to ensure smooth operation and integration of the new technology. This comprehensive training and

resource allocation aimed to facilitate the transition to a digital system and improve the overall management of the graveyards.

Despite the intensive efforts to train and support digital technology use, two supervisors refused to receive the training and did not adopt the digital death record-keeping system.

# Appendix 7: Digital death record keeping and management systems for the graveyards.

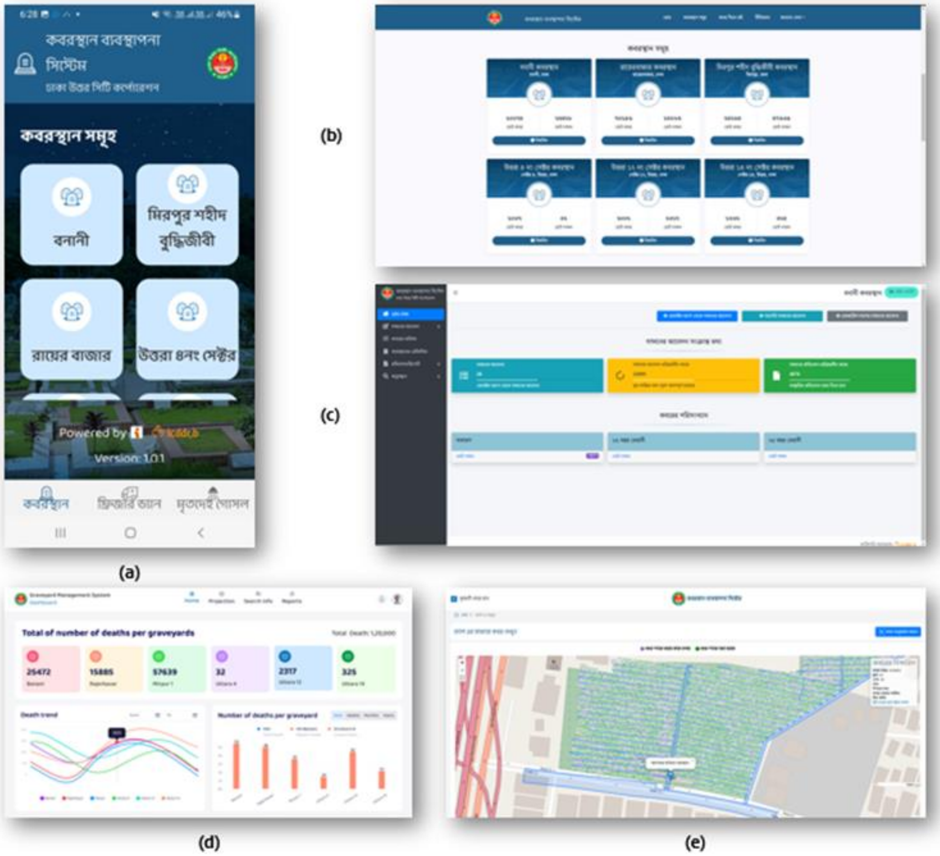


# Appendix 8: Details of System Usability Scale (SUS) and Technology Acceptance Model (TAM)

SUS is widely utilized to evaluate perceived usability in usability-related studies (1, 2). Previous studies have demonstrated that SUS exhibits a significant level of reliability, typically with a Cronbach's alpha coefficient exceeding 0.90 (3). Additionally, the SUS has been found to possess validity and may be customized to suit various settings (3). The SUS consists of a total of 10 elements, with half of them exhibiting a positive tone (the odd-numbered items) and the remaining half displaying a negative tone (the even-numbered things). The response is provided on a numerical scale ranging from 1 (indicating strong disagreement) to 5 (indicating strong agreement) for each item. The SUS score goes from 0 to 100, with higher scores indicating better usability. The total System Usability Scale (SUS) score was calculated using the following formula (4): The positive statement item score was reduced by 1 (x-1), whereas the negative

statement item score was obtained by subtracting the score from 5 (5-x). The total of these item scores was subsequently multiplied by a factor of 2.5 to yield an overall SUS score ranging from 0 (indicating extremely low usability) to 100 (indicating exceptional usability).

Similar to SUS, the response for the TAM items is also given on a scale of 1 (strongly disagree) to 5 (strongly agree). Perceived Usefulness (PU) and Perceived Ease of Use (PEOU) are the two core constructs of TAM that have 6 items each for the respective measurements. In the context of Technology Acceptance Model (TAM), PU stands for the extent to which an individual believes that utilizing technology would enhance their job performance, as defined by (5). PEOU, or Perceived Ease of Use, is the extent to which an individual believes that utilizing technology would be effortless and devoid of any exertion (5). However, in a formal sense, the Technology Acceptance Model (TAM) introduces the notions of Perceived Usefulness (PU) and Perceived Ease of Use (PEOU) as characteristics that influence user acceptance before actually using a product or technology. There is a positive correlation between a person's belief in these elements and their intention to use them. The stronger the belief, the higher the intention to use. The initial findings of the Technology Acceptance Model (TAM) study conducted by Davis and colleagues (5) indicated a strong and statistically significant correlation between both Perceived Usefulness (PU) and Perceived Ease of Use (PEOU) with users' inclination to anticipate using a product if it were made accessible to them. The PU (Perceived Usefulness) and PEOU (Perceived Ease of Use) sub-dimensions had a high level of reliability, with coefficients of 0.98 and 0.94 respectively. Additionally, these sub-dimensions exhibited appropriate levels of convergent and divergent validities (6). Supplementary material ## presents the 14 items associated with PU (Perceived Usefulness) and PEOU (Perceived Ease of Use) according to the original version of the TAM (Technology Acceptance Model) (5).

## References


1. Lewis JR. Usability: Lessons Learned … and Yet to Be Learned. International Journal of Human–Computer Interaction. 2014;30(9):663-84.

2. Lewis JR. The System Usability Scale: Past, Present, and Future. International Journal of Human–Computer Interaction. 2018;34(7):577-90.

3. Peres SC, Pham T, Phillips R. Validation of the System Usability Scale (SUS): SUS in the Wild. Proceedings of the Human Factors and Ergonomics Society Annual Meeting. 2013;57(1):192-6.

4. Brooke J. SUS-A quick and dirty usability scale. Usability evaluation in industry. 1996;189(194):4-7.

5. Davis FD. Perceived usefulness, perceived ease of use, and user acceptance of information technology. MIS quarterly. 1989:319-40.

6. Pal D, Vanijja V. Perceived usability evaluation of Microsoft Teams as an online learning platform during COVID-19 using system usability scale and technology acceptance model in India. Children and Youth Services Review. 2020;119:105535.


## System Usability Scale (SUS)

| SL | Questionnaire | Strongly Disagree | | Neutral | Strongly Agree | |
|---|---|---|---|---|---|---|
| 201 | I think that I would like to use this website, apps and digital deaths records keeping system. | 1 | 2 | 3 | 4 | 5 |
| 202 | I found the Graveyard website, apps and digital deaths records keeping system unnecessarily complex. | 1 | 2 | 3 | 4 | 5 |
| 203 | I thought the graveyard website, apps and digital deaths records keeping system was easy to use. | 1 | 2 | 3 | 4 | 5 |
| 204 | I think that I would need the support of a technical person to be able to use this graveyard website, apps and digital deaths records keeping system. | 1 | 2 | 3 | 4 | 5 |
| 205 | I found the various functions in this graveyard website, apps and digital deaths records keeping system were well integrated. | 1 | 2 | 3 | 4 | 5 |
| 206 | I thought there was too much inconsistency in this graveyard website, apps and digital deaths records keeping system. | 1 | 2 | 3 | 4 | 5 |
| 207 | I imagine that most people would learn to use this graveyard website, apps and digital deaths records keeping system very quickly. | 1 | 2 | 3 | 4 | 5 |
| 208 | I found this graveyard website, apps and digital deaths records keeping system very awkward to use. | 1 | 2 | 3 | 4 | 5 |
| 209 | I felt very confident using this graveyard website, apps and digital deaths records keeping system. | 1 | 2 | 3 | 4 | 5 |
| 210 | I needed to learn a lot of things before I could get going with this graveyard website, apps and digital deaths records keeping system. | 1 | 2 | 3 | 4 | 5 |

**Technology Acceptance Model (TAM)**

| SL | **Questionnaire Items of TAM** | Strongly Disagree | Neutral | Strongly Agree |
|---|---|---|---|---|

| | | | | | | |
|---|---|---|---|---|---|---|
| 301. | Using this graveyard website, apps and digital deaths records keeping system. will enable me to accomplish submission of burial application, knowing information about burial, knowing information about graveyards and search information about burial person more quickly. | 1 | 2 | 3 | 4 | 5 |
| 302. | Using this graveyard website, apps and digital deaths records keeping system. will improve process of submission of burial application, knowledge about burial information, knowledge about graveyard information and search information about burial person. | 1 | 2 | 3 | 4 | 5 |
| 303. | Using this graveyard website, apps and digital deaths records keeping system will increase productivity for submission of burial application, knowledge about burial information, knowledge about graveyard information and search information about burial person. | 1 | 2 | 3 | 4 | 5 |
| 304. | Using this graveyard website, apps and digital deaths records keeping system will enhance the submission of burial application, knowledge about burial information, knowledge about graveyards, and facilitate searching for information about the deceased person. | 1 | 2 | 3 | 4 | 5 |
| 305. | Using this graveyard website, apps and digital deaths records keeping system will make the submission of burial application easier, provide knowledge about burial information, provide information about graveyards, and facilitate the search for information about the deceased person. | 1 | 2 | 3 | 4 | 5 |

# APPENDIX 9: DATA COLLECTION TOOLS FOR IDIS

Interview Questions:

1. Acceptability
   - How do you feel about using the digital death record-keeping system?
   - Can you describe any initial reactions you had to the system?
2. Adoption
   - Have you adopted the digital death record-keeping system? If yes, how was the transition from paper to digital for you?
   - If no, what are the reasons for not adopting the system?
3. Appropriateness
   - Do you think the digital system is suitable for your work? Why or why not?
   - In what ways has the digital system improved or hindered your tasks?
4. Feasibility
   - How easy or difficult do you find using the digital death record-keeping system?
   - Do you have the necessary resources (e.g., computers, internet) to use the system effectively?
5. Barriers
   - What challenges have you faced while using the digital system?
   - Are there any specific aspects of the system that you find particularly difficult or frustrating?
6. Sustainability
   - Do you believe the digital death record-keeping system can be sustained in the long term? Why or why not?
   - What support or resources would help ensure the sustainability of the system?
